# Combined use of CSF NfL and CSF TDP‐43 improves diagnostic performance in ALS

**DOI:** 10.1002/acn3.50943

**Published:** 2019-11-19

**Authors:** Takashi Kasai, Yuta Kojima, Takuma Ohmichi, Harutsugu Tatebe, Yukiko Tsuji, Yu‐ichi Noto, Fukiko Kitani‐Morii, Makiko Shinomoto, David Allsop, Toshiki Mizuno, Takahiko Tokuda

**Affiliations:** ^1^ Department of Neurology Kyoto Prefectural University of Medicine Kyoto 602‐0841 Japan; ^2^ Department of Medical Innovation and Translational Medical Science Kyoto Prefectural University of Medicine Kyoto 602‐0841 Japan; ^3^ Division of Biomedical and Life Sciences Faculty of Health and Medicine Lancaster University Lancaster LA1 4YQ United Kingdom; ^4^ Department of Molecular Pathobiology of Brain Diseases Kyoto Prefectural University of Medicine Kyoto 602‐0841 Japan

## Abstract

**Objective:**

To determine the diagnostic and prognostic significance of neurofilament light chain (NfL), TAR DNA‐binding protein 43 (TDP‐43), and total tau (t‐tau) in cerebrospinal fluid (CSF) and plasma of patients with amyotrophic lateral sclerosis (ALS) and to investigate whether the combined use of those biomarker candidates can improve their diagnostic performance.

**Methods:**

This was a single–center, prospective, longitudinal study. CSF and plasma samples were collected at the time of enrollment from a discovery cohort of 29 patients with ALS and 29 age–matched controls without neurodegenerative disease. In a validation cohort, there were 46 patients with ALS, and 46 control (not age‐matched) patients with motor weakness resulting from neuromuscular diseases. NfL, TDP‐43, and t‐tau levels in CSF and plasma were measured using ultrasensitive single molecule assay (Simoa) technology.

**Results:**

The following findings were reproducibly observed among the discovery and validation cohorts: increased levels of CSF NfL, plasma NfL, and CSF TDP‐43 in ALS compared with control groups; shorter survival associated with higher levels of CSF and plasma NfL. When the CSF NfL and CSF TDP‐43 levels were combined, the areas under the ROC curves (AUC) were slightly improved relative to AUCs for each biomarker alone.

**Interpretation:**

CSF and plasma NfL may not only serve as diagnostic biomarkers but also provide a measure of disease progression. CSF TDP‐43 is also useful as a diagnostic biomarker of ALS, but has no prognostic value. The combined use of CSF NfL and CSF TDP‐43 may be a useful biomarker for the diagnosis of ALS.

## **Introduction**

The most promising biomarker for amyotrophic lateral sclerosis (ALS) is neurofilament light chain (NfL) at present. Elevated levels of NfL in cerebrospinal fluid (CSF) and plasma/serum have been reported in patients with ALS compared with controls; moreover, they were associated with poor outcomes.^1^, ^2^ TAR DNA‐binding protein 43 (TDP‐43) positive inclusions are found in approximately 97% of patients with ALS. This has led to the investigation of TDP‐43 as a potential molecular biomarker for ALS. Overall, these studies have identified increased levels of TDP‐43 in CSF from ALS patients compared with controls.^3^ An elevated level of TDP‐43 has also been reported in plasma from ALS patients in one case–control study.^4^ However, the absolute concentrations of TDP‐43 in CSF and plasma have varied across studies, suggesting that TDP‐43 immunoassays are inconsistent for measuring this protein within biofluids.^3^ The other candidate is Tau. Recent studies reporting elevated levels of CSF total Tau (t‐tau) in ALS patients compared with controls have generated novel interest in the diagnostic potential of t‐tau for ALS.^5^, ^6^ However, there are conflicting results, which ranged between normal^5^, ^6^ and increased levels,^7^, ^8^ and the prognostic significance of plasma t‐tau in ALS has so far received little attention. Moreover, it still needs to be elucidated whether the combined use of the biomarker candidates can improve their diagnostic performance for ALS because of the lack of comprehensive analysis of those three molecules. Thus, we conducted this study to determine the diagnostic and prognostic potential of TDP‐43 and t‐tau as molecular biomarkers, compared with NfL not only in CSF but also in plasma.

## Methods

### Study design, ethics approvals, and subject recruitment

The study was a hospital‐based case‐control study. All study subjects provided written informed consent before participation and the study protocols were approved by the University Ethics Committee (ERB‐G‐12). Informed consent from patients was obtained when possible and also from the nearest relative. Study procedures were designed and performed in accordance with the Declaration of Helsinki. The discovery cohort consisted of 29 individuals with possible, probable, or definite ALS and 29 age–matched controls. The control group participants had nonneurodegenerative diseases and presented with no neurological symptoms. All participants of the discovery cohort underwent CSF and plasma collection. The sample size of the discovery cohort was set according to the effect size of previous biomarker studies.^9^, ^10^ The validation cohort comprised 46 individuals with suspected, possible, probable, or definite ALS and 46 patients with motor weakness resulting from neuromuscular diseases, comprising: chronic inflammatory demyelinating polyneuropathy (CIDP: *N* = 17), Guillain–Barre syndrome (GBS: *N* = 18), multifocal motor neuropathy (MMN: *N* = 6), and inclusion body myositis (IBM: *N* = 5). (See Data S1 for diagnostic criteria for those diseases) Of note, not all participants in the validation cohort provided both blood and CSF samples. (Plasma samples were not collected from 26 participants. CSF samples of 5 participants were unavailable.) Because relatively young individuals were included in the control group, the ALS and control groups were not age‐matched in the validation. The participants of the discovery and validation cohorts were enrolled from the registration in our institute from September 2009 to March 2014 and from April 2014 to May 2018, respectively. ALS patients in the both cohorts were diagnosed according to the revised El Escorial criteria.^11^ All suspected and possible ALS patients were confirmed to show conversion to probable or definite ALS within the follow‐up period. The follow‐up period was defined as the period (days) from the date of sample collection to the date of any endpoint or right‐censoring (when a subject left the study before an event, or the study ended prior to an event).

### Measurement

All measurements of the biomarkers were carried out on a Simoa HD‐1 Analyzer (Quanterix, Lexington, MA) using of commercially available kits.

For detailed information of measurements, sample preparation, and statistical analyses, see Data S1.

## Results

### Patient characteristics

The demographic characteristics are summarized in Table 1 (for clinical information and raw data on biomarker concentrations, see Tables S1 and S2) There was no significant difference in age (*P* = 1.000) or sex (*P* = 0.7840) between the ALS and control groups in the discovery cohort. In the validation cohort, the median age of the control group was significantly younger than that of the ALS group (*P* < 0.0001), while there was no significant difference in sex between the two groups (*P* = 0.3696).

**Table 1:** Patients characteristics.

Category	Specific diagnosis	*N*	Sex(M:F)	Age
The discovery cohort				
ALS		29	18:11	65.41 ± 12.34
Control (nonneurodegenerative control)	See Table S1B	29	19:10	66.40 ± 9.2
	Difference between the groups:		*P* = 1.000	*P* = 0.7840
The validation cohort				
ALS		46	29:17	71.36 ± 9.27
Control (patients with motor weakness from neuromuscular diseases)		46	34:12	69.83 ± 20.18
	Difference between the groups:		*P* = 0.3696	*P* < 0.0001
	CIDP	17	14:3	60.06 ± 14.45
	GBS	18	11:7	50.67 ± 23.80
	MMN	6	5:1	48.50 ± 21.03
	IBM	5	4:1	76.00 ± 2.45

GBS, Gullain–Barre syndrome; CIDP, chronic inflammatory demyelinating polyneuropathy; MMN, multifocal motor neuropathy; IBM, inclusion body myositis.

### Concentrations of biomarkers in the discovery cohort

**Figure 1:**
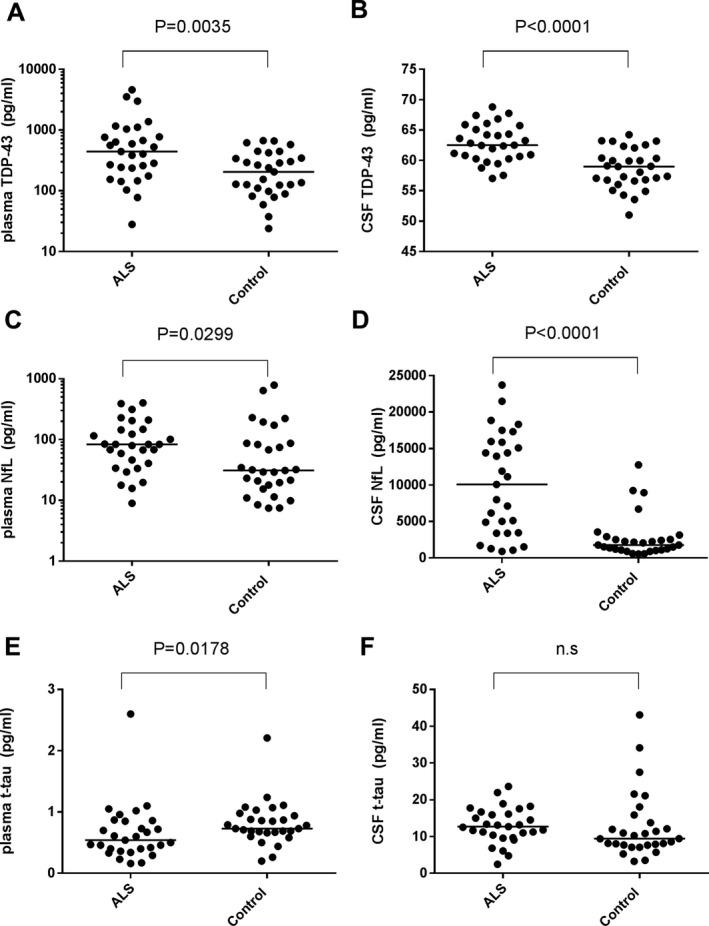
Scatter plots of biomarker levels in the discovery cohort. ALS (*n* = 29) and control (*n* = 29). Levels of plasma and CSF TDP‐43 (A, B), NfL (C, D), t‐tau (E, F) are presented. Bars indicate median values. The *P*‐value generated by Mann–Whitney’s *U* test is shown above each graph. n.s.: not significant. Note: The vertical axes of plasma TDP‐43 (A) and plasma NfL (C) use a logarithmic scale.

The concentrations of TDP‐43, NfL, and t‐tau in the samples from the discovery cohort are summarized in Figure 1. In the case of TDP‐43, both plasma (*P* = 0.0035, Fig. 1A) and CSF levels (*P* < 0.0001, Fig. 1B) of this marker were elevated in the ALS group compared with the control group. This was also the case for NfL with increased levels found in both plasma (*P* = 0.0299, Fig. 1C) and CSF (*P* < 0.0001, Fig. 1D) from the ALS group compared with the control group. Finally, t‐tau levels were significantly lower in the ALS group only in plasma (*P* = 0.0178, Fig. 1E), and not in CSF (*P* = 0.1062, Fig. 1F).

### ROC analysis of biomarkers in the discovery cohort

According to ROC analysis of the discovery cohort, CSF NfL generated the highest area under the curve (AUC) value (AUC = 0.8347, Fig. S1D). The second highest AUC value was observed with CSF TDP‐43 (AUC = 0.8205, Fig. S1B).

### Correlation between levels of biomarkers in CSF and plasma in the discovery cohort

There was a significant positive correlation between NfL levels of plasma and CSF taken from each patient with ALS in the discovery cohort (solid line, *P* < 0.0001). Such a significant CSF‐plasma correlation was also identified in the control group (dashed line, *P* = 0.0013) (Fig. S2B). Neither TDP‐43 nor t‐tau levels showed any plasma‐CSF correlation in either of the groups (TDP‐43 in the ALS group: *P* = 0.2279, TDP‐43 in the control group: *P* = 0.9252, t‐tau in the ALS group: *P* = 0.1024, t‐tau in the control group: *P* = 0.3463) (Fig. S2A and C).

### Biomarkers and survival times in the discovery cohort

**Figure 2:**
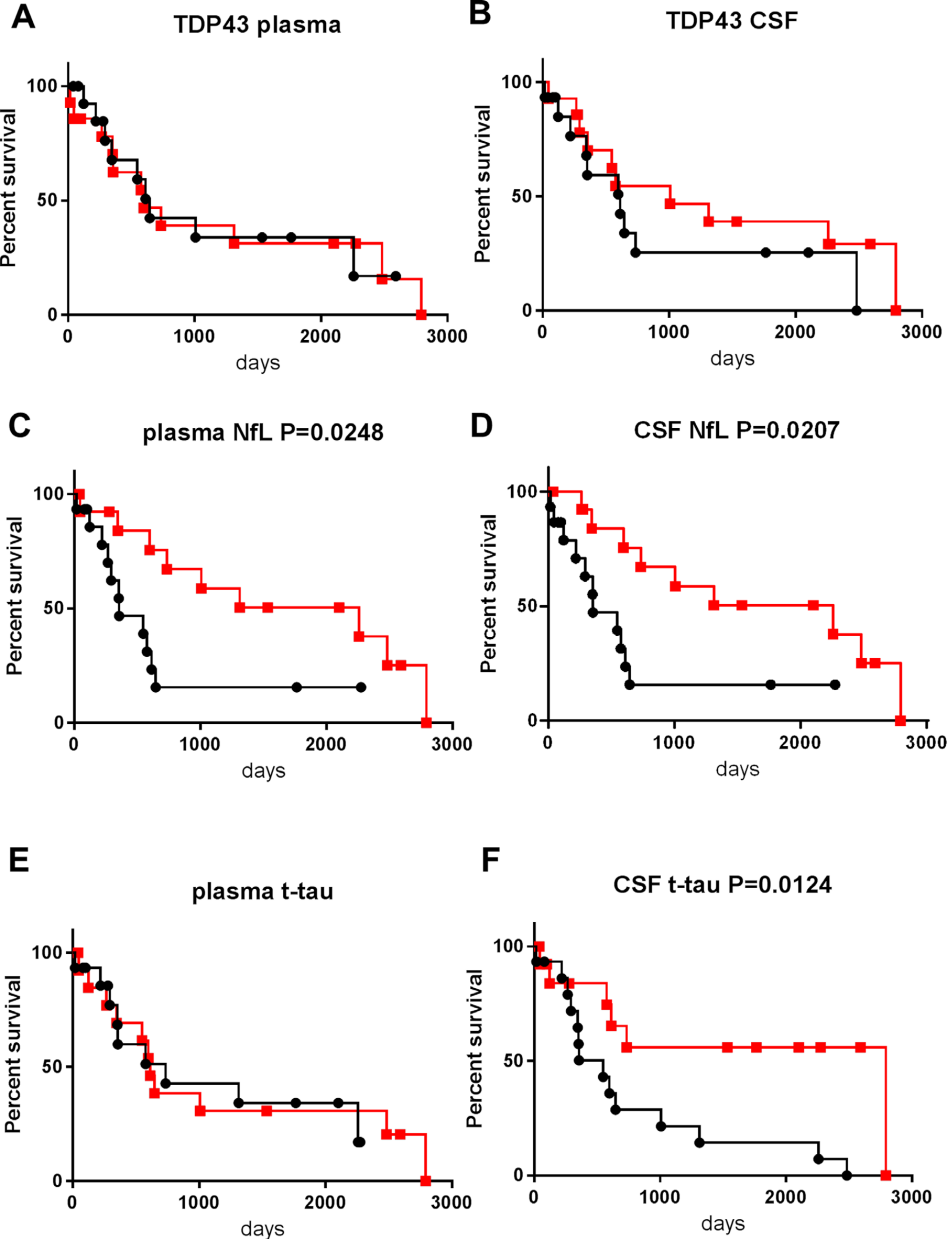
Kaplan–Meier survival curves in ALS patients of the discovery cohort according to biomarkers levels. (A): plasma TDP‐43, (B): CSF TDP‐43, (C): plasma NfL, (D): CSF NfL, (E): plasma t‐tau, (F): CSF t‐tau. The squares and circles indicate an event (death, tracheostomy, or invasive ventilation). Patients were subdivided into two groups according to the cut‐off biomarker levels. The cut‐off value in each graph was set as the median value of the corresponding biomarker within the ALS group. The black lines with black circles represent patients with levels of biomarkers no lower than the cut‐off (the high–level group). The red lines with red squares represent those with levels lower than the cut‐off (the low–level group).

All members of the ALS group in the discovery cohort were included in log‐rank analysis (Fig. 2). Nineteen patients reached the endpoint of death, tracheostomy, or invasive ventilation during the follow‐up period. Survival times ranged from 17 to 2793 days (median: 575 days) (Table S1B). Patients with ALS were subdivided into two groups according to the levels for each of the biomarkers: a low–level group (<median value), and a high–level group (≥median value). When comparing the high– and low–level groups, significant differences were noted in plasma NfL (*P* = 0.0248, Fig. 2C), CSF NfL (*P* = 0.0207, Fig. 2D), and CSF t‐tau (*P* = 0.0124, Fig. 2F), while there is no significant difference in plasma TDP‐43, CSF TDP‐43, or plasma t‐tau (Fig. 2A, B, E). The high–level groups were associated with shorter survival compared with the low–level groups, for plasma NFL, CSF NfL, and CSF t‐tau. After age‐adjustment in multivariate analysis, the high levels of plasma and CSF NfL still retained significant prognostic value (plasma NfL, Hazard ratio (HR) = 6.800, *P* = 0.003; CSF NfL, HR = 7.727, *P* = 0.002), while the association between CSF t‐tau and survival did not reach significance (CSF t‐tau, HR = 2.875, *P* = 0.065).

### Concentrations of biomarkers in the validation cohort

**Figure 3:**
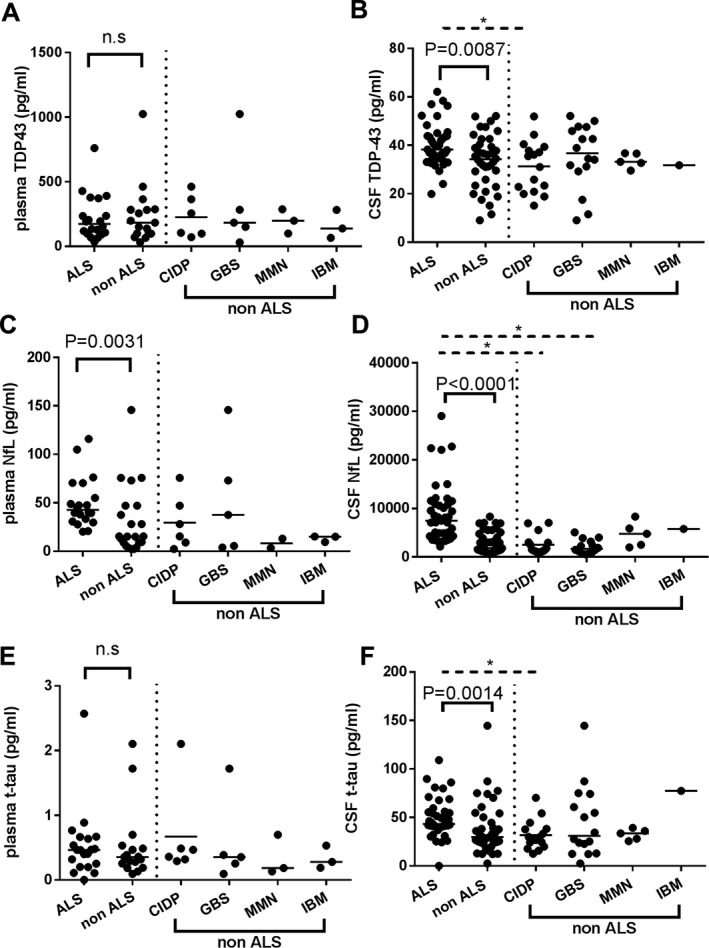
Scatter plots of biomarker levels in the validation cohort. Control (*n* = 46) and ALS (*n* = 46). Levels of plasma and CSF TDP‐43 (A, B), NfL (C, D), t‐tau (E, F) are presented. Bars indicate median values. The *P*‐value generated by Mann–Whitney’s *U* test between the ALS and whole control group is shown above each graph. Significant differences were reproducibly confirmed by multiple comparison tests with the Kruskal–Wallis test among the ALS group and subgroups of the controls (CIDP, GBS, MMN, and IBM). Dashed bars and asterisks indicate significant differences (*P* < 0.05) between the groups by post hoc analysis of Dunn’s multiple comparison procedure. n.s.: not significant.

The concentrations of TDP‐43, NfL, and t‐tau in the validation cohort are summarized in Figure 3. On comparing ALS and control groups, significant elevations of biomarker concentrations in the ALS group were reproduced for CSF TDP‐43 (*P* = 0.0087, Fig. 3B), plasma NfL (*P* = 0.0031, Fig. 3C), and CSF NfL (*P* < 0.0001, Fig. 3D), while neither plasma TDP‐43 nor plasma t‐tau levels were different between the groups, in contrast to those in the discovery cohort. CSF t‐tau levels in the ALS group were significantly higher than those in the control group in the validation cohort, although such a difference was not observed in the discovery cohort. Those significant differences were reproducibly confirmed by multiple comparison with the Kruskal–Wallis test among the ALS group and subgroups of the controls (CIDP, GBS, MMN, and IBM). Post hoc analysis of Dunn’s multiple comparison tests revealed significantly higher levels of CSF TDP‐43 in the ALS group compared with those in the CIDP subgroup, CSF NfL in the ALS group compared with those in the CIDP and GBS subgroups, and CSF t‐tau in the ALS group compared with those in the CIDP subgroup. The ROC analyses are summarized in Figure S3. Considering the age difference between the ALS and control groups, we reanalyzed those comparisons after the exclusion of individuals younger than 60 years of age (Fig. S4). There was no significant difference in age between the ALS (*n* = 42) and control (*n* = 24) groups, consisting of individuals aged no younger than 60. In these advanced age groups, comparisons between groups regarding biomarkers showing significant differences between the groups based on raw data (CSF TDP‐43, CSF NfL, plasma NfL, and CSF t‐tau) were conducted. Significant elevation of CSF TDP‐43 and CSF NfL and plasma NfL levels in the ALS group compared with those in controls was preserved (*P* = 0.004 in Fig. S4A, *P* = 0002 in Fig. S4B, and *P* = 0.0156 in Fig. S4C, respectively), while the difference between the groups regarding CSF t‐tau did not reach significance (Fig. S4D).

### Biomarkers and survival times in the validation cohort

**Figure 4:**
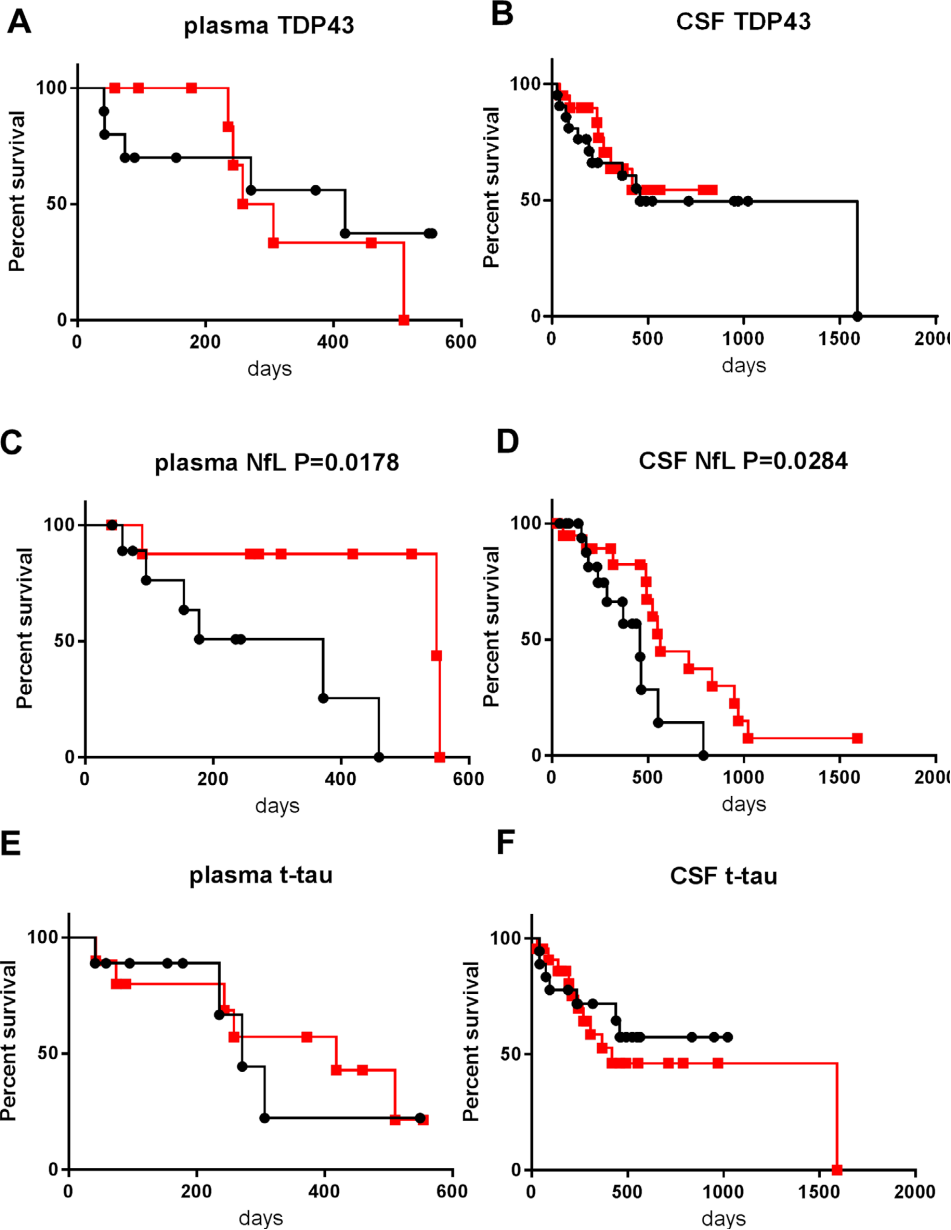
Kaplan–Meier survival curves in ALS patients of the validation cohort according to biomarker levels. (A): plasma TDP‐43, (B): CSF TDP‐43, (C): plasma NfL, (D): CSF NfL, (E): plasma t‐tau, (F): CSF t‐tau. Patients were subdivided into two groups according to the cut‐off biomarker levels. The cut‐off value in each graph was set as the median value of the corresponding biomarker within the ALS group. The squares and circles indicate an event (death, tracheostomy, or invasive ventilation). The black lines with black circles represent patients with levels of biomarkers no lower than the cut‐off (the high–level group). The red lines with red squares represent those with levels lower than the cut‐off (the low–level group).

Not all patients with ALS in the validation cohort were included in the log‐rank analysis due to missing samples. We performed survival analysis involving 20 ALS patients with plasma biomarker data and 41 ALS patients with CSF biomarker data (Fig. 4). In those patients, 10 patients in plasma biomarker analysis and 18 patients in CSF biomarker analysis reached the endpoint. Survival times ranged from 28 to 1592 days (median: 305 days) (Table S2B). The high–level group showed significantly shorter survival compared with the low–level group for plasma NfL (*P* = 0.0178, Fig. 4C) and CSF NfL (*P* = 0.0284, Fig. 4D), corresponding with the results in the discovery cohort. However, the significant difference in CSF t‐tau was not reproduced (Fig. 4F). After age‐adjustment, the high levels of plasma and CSF NfL still exhibited significant prognostic values (HR = 12.262, *P* = 0.041 and HR = 4.83, *P* = 0.01, respectively).

### ROC analysis of composite biomarkers in discovery and validation cohorts regarding CSF TDP‐43, CSF NfL and plasma NfL

**Figure 5:**
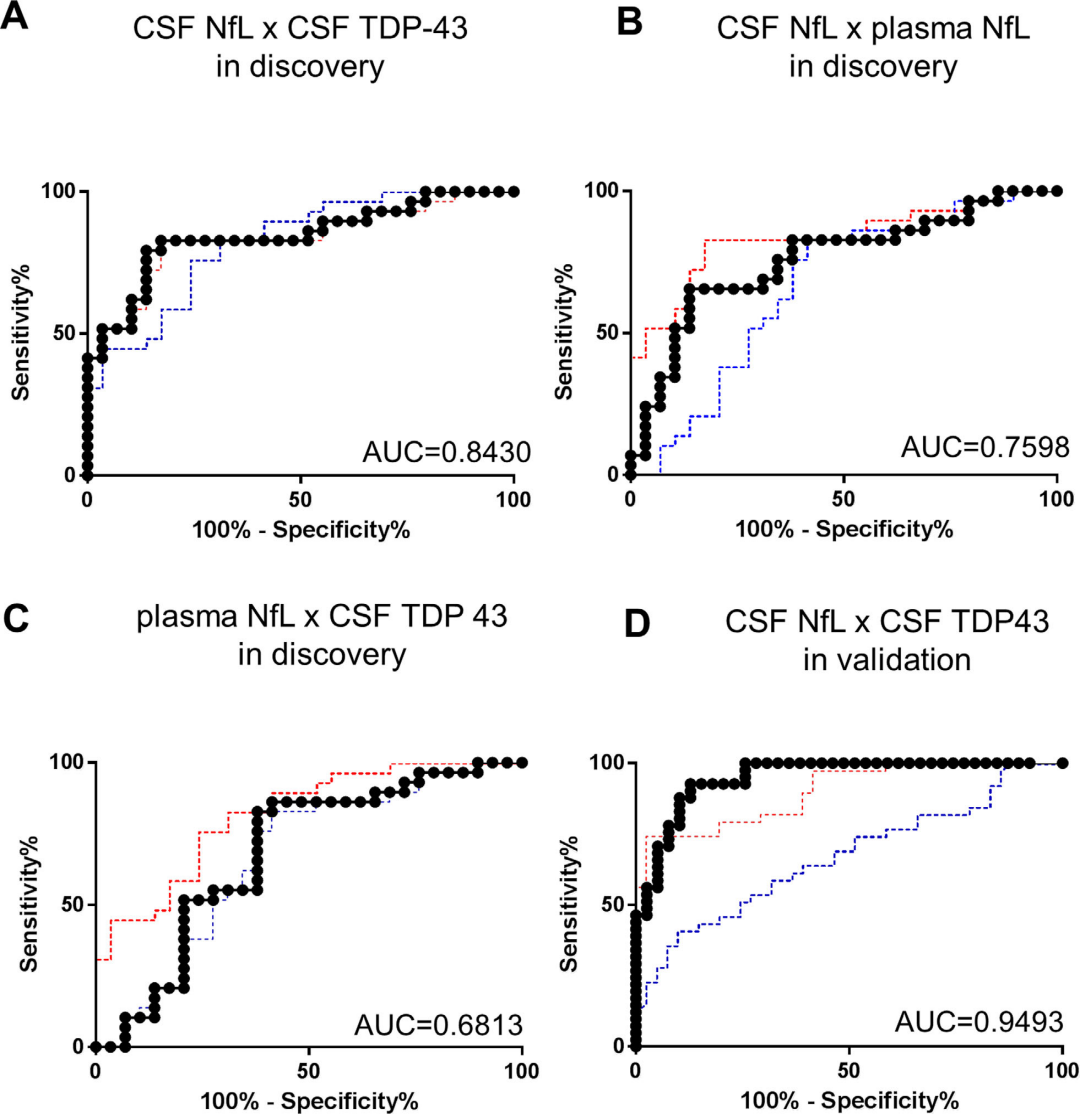
Receiver operating characteristic (ROC) analyses for the composite parameters of the discovery and validation cohorts. AUC values are indicated in the graphs. The title of each graph represents the biomarker used as an independent variable on analysis: (A) the products of CSF NfL and CSF TDP‐43 in the discovery cohort. The red and blue dotted lines respectively indicate the ROC curves of CSF NfL alone and CSF TDP 43 alone for reference (see Figs. S1 and S4 regarding the ROC analyses of each biomarker for details.) (B) the products of plasma NfL and CSF NfL in the discovery cohort. The red and blue dotted lines respectively indicate the ROC curves of CSF NfL alone and plasma NfL alone. (C) the products of plasma NfL and CSF TDP‐43 in the discovery cohorts; the red and blue dotted lines respectively indicate the ROC curves of CSF TDP‐43 alone and plasma NfL alone. (D) the products of CSF NfL and CSF TDP‐43 in the validation cohort; the red and blue dotted lines respectively indicate the ROC curves of CSF NfL alone and TDP‐43 alone. Sensitivity/specificity of the products of CSF NfL × CSF TDP‐43, CSF TDP‐43 alone, CSF NfL alone, and plasma NfL alone in the discovery cohort reached 79.31%/86.21%, 75.86%/75.86%, 82.76%/82.76%, and 62.07%/65.52%, respectively. Those in the validation cohort were 87.80%/89.74% for the products of CSF NfL × CSF TDP‐43, 73.17% /53.85% for CSF TDP‐43 alone, and 80.49%/74.36% for CSF NfL alone, respectively.

Regarding the CSF TDP‐43, CSF NfL, and plasma NfL markers that showed significant elevation in the ALS compared with control groups for both discovery and validation cohorts, we calculated composite parameters of the products of CSF NfL × CSF TDP‐43, of CSF NfL × plasma NfL, and of plasma NfL × CSF TDP‐43 (Fig. 5). In both cohorts, the combination of CSF NfL and CSF TDP‐43 provided better performance in terms of the AUC value (AUC = 0.8430 and 0.9493 in the discovery and validation cohorts, respectively) compared to those for each biomarker alone. The discriminability for the product of CSF NfL × plasma NfL (AUC = 0.7598) was inferior to that of CSF NfL alone, in the discovery cohort. The AUC value for the combination of plasma NfL and CSF TDP‐43 (0.6813) did not exceed that for CSF TDP‐43 alone. Combined analyses for the CSF and plasma biomarkers were not performed in the validation cohort because more than half of the participants of this cohort did not undertake both plasma and CSF collection. (See Fig. S5 regarding ROC analysis for optimally weighted composite biomarkers.)

### Combined analysis of validation and discovery cohorts regarding plasma TDP‐43, CSF TDP‐43, plasma t‐tau, and CSF t‐tau

**Figure 6:**
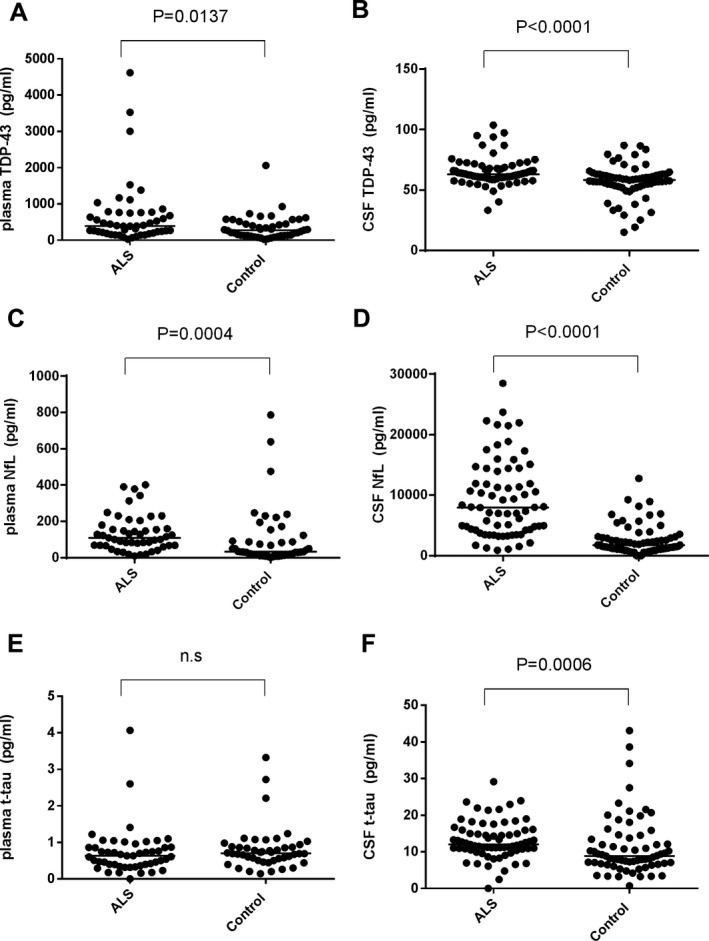
Scatter plots of biomarker levels in combined analysis of the discovery and validation cohorts. Analyses of plasma biomarkers involved 49 ALS patients and 47 controls; CSF biomarker analyses involved 71 ALS patients and 68 controls. Levels of plasma and CSF TDP‐43 (A, B), NfL (C, D), t‐tau (E, F) are presented. Because of interassay variation, we corrected the values of the validation cohort based on the correction formula: raw values × correction coefficient: 2.01 for plasma TDP‐43, 1.67 for CSF TDP‐43, 3.26 for plasma NfL, 0.98 for CSF NfL, 1.58 for plasma t‐tau, and 0.27 for CSF t‐tau. The correction factors were determined as the mean value ratios between the discovery and validation assays based on four internal controls for each biomarker. Bars indicate median values. The *P*‐value generated by Mann–Whitney’s *U* test between the ALS and whole control groups is presented above each graph. n.s: not significant. Note: The correction coefficients were higher than those expected from interassay concordance reported by the supplier. This may be attributable to lot‐to‐lot differences of the kits (see Table S3).

**Figure 7:**
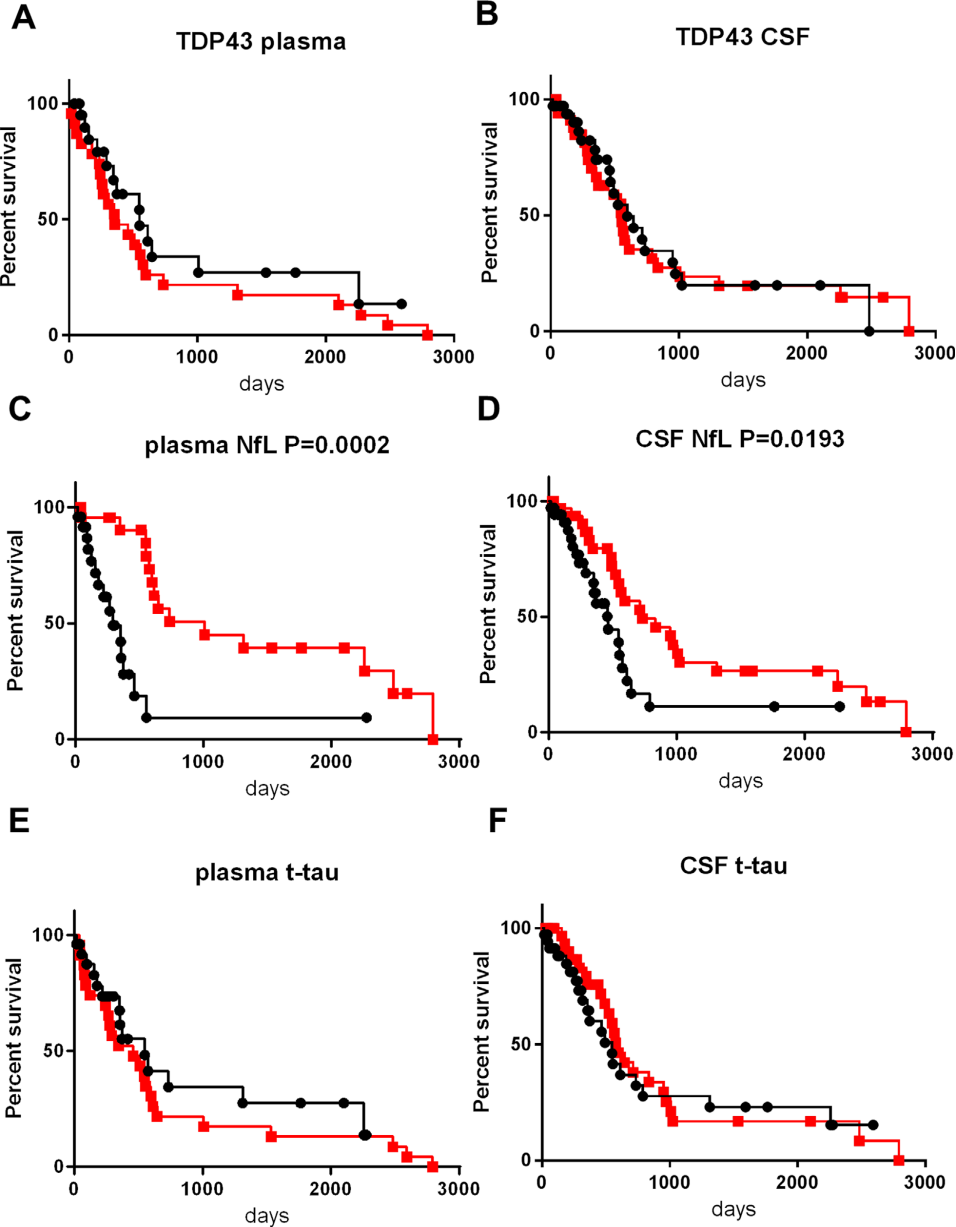
Kaplan–Meier survival curves in ALS patients on combined analysis of the discovery and validation cohorts. Correction of interassay variation was conducted using the formula presented in Figure 6. (A): plasma TDP‐43, (B): CSF TDP‐43, (C): plasma NfL, (D): CSF NfL, (E): plasma t‐tau, (F): CSF t‐tau. Patients were subdivided into two groups according to the cut‐off biomarker levels. The cut‐off value in each graph was set as the median value of the corresponding biomarker within the ALS group. The squares and circles indicate an event (death, tracheostomy, or invasive ventilation). The black lines with black circles represent patients with levels of biomarkers no lower than the cut‐off (the high–level group). The red lines with red squares represent those with levels lower than the cut‐off (the low–level group).

Regarding the levels of plasma TDP‐43, plasma t‐tau, and CSF t‐tau, for which inconsistent differences were found between ALS patients and controls when comparing the two cohorts, we conducted a combined analysis based on data from internal controls (Fig. 6). Levels of plasma TDP‐43 in the combined ALS group were higher than those in the combined control group (*P* = 0.0137, Fig. 6A). Levels of plasma t‐tau were not different between these groups (*P* = 0.228, Fig. 6E), while CSF t‐tau was significantly elevated in the combined ALS group compared with the combined control group (*P* = 0.0006, Fig. 6F). We compared the biomarker levels regarding disease duration (within 10 months vs. more than 10 months from onset), onset area (bulbar onset vs. others), and dementia status in the ALS group, no difference was observed (data not shown). We also recalculated survival analyses in the combined ALS group for the biomarkers. Both plasma and CSF NfL levels were associated with shorter survival (*P* = 0.0002 and *P* = 0.0193, respectively). Those significances were still preserved after age‐adjustment (HR = 7.611, *P* < 0.001 and HR = 4.567, *P* < 0.001, respectively). Meanwhile, there was no significant difference in survival between the high– and low–level groups based on TDP‐43 and t‐tau levels in plasma and CSF (Fig. 7).

## Discussion

Biomarker profiles of TDP‐43, NfL, and t‐tau in ALS have been individually well‐investigated.^3^ However, most previous studies have focused on one or two of these biomarkers. Moreover, the diagnostic or prognostic value of plasma TDP‐43 or plasma t‐tau in ALS has remained uncertain because of the difficulty of stable measurement. To the best of our knowledge, this study is the first to comprehensively measure levels of all of these three candidate biomarkers, not only in CSF but also, simultaneously, in plasma. This study showed the following three major findings that were consistent across the discovery and validation cohorts.

First, CSF NfL was significantly elevated in the ALS compared with control groups. Furthermore, the potential prognostic value of elevated levels of CSF NfL, in terms of shorter survival time, was observed after stratifying cohorts according to the median CSF NfL levels. These confirm the findings gathered in retrospective case–control studies and prospective observations.^1^, ^2^, ^12^, ^13^, ^14^, ^15^, ^16^, ^17^

Second, plasma NfL was significantly higher in the ALS group than in the controls, and higher plasma NfL was associated with a shorter survival. These results are in agreement with observations in previous case–control studies using serum^16^, ^18^, ^19^ and plasma.^13^ Overall, these findings support the possibility that NfL not only in CSF but also plasma, can serve as a promising biomarker for the diagnosis and monitoring of disease progression of ALS. The fact that CSF and plasma NfL shared the same biomarker profile is reasonable when we consider the correlation between them in each participant of the discovery cohort.

Finally, we noted significantly higher levels of TDP‐43 in the CSF of ALS patients than those in controls. This result is consistent with previous observations, including two of our studies and one meta‐analysis.^9^, ^10^, ^20^, ^21^, ^22^, ^23^ TDP‐43 is considered to be a disease–specific biomarker reflecting TDP‐43 pathology in the central nervous system. As expected, the AUC values representing the ability to discriminate between ALS patients and controls were improved by combining CSF NfL with CSF TDP‐43 relative to that of each biomarker alone. This observation was found consistently across both cohorts, suggesting that CSF TDP‐43 could serve as a biomarker complementary to NfL, for the diagnosis of ALS. CSF NfL was reported recently to have a diagnostic potential even for presymptomatic ALS.^18^ However, at present, it is not possible to predict which kind of neurodegeneration will develop in individuals with elevated CSF NfL levels due to its lack of disease specificity.^24^ The combined use of CSF NfL and CSF TDP‐43 may be recommended for such people suspected to have neurodegeneration with undetermined pathology.

Levels of plasma TDP‐43, plasma t‐tau, and CSF t‐tau were significantly different between the ALS and control groups in either the discovery or validation cohort, although the results were not preserved across these cohorts. In the combined analysis, significant elevation of plasma TDP‐43 and CSF t‐tau in the ALS group was repeatedly observed, whereas the significant difference in plasma t‐tau between the groups was not reproduced. Considering those facts, levels of plasma TDP‐43 and CSF t‐tau may be likely increased in ALS compared to controls although future validation studies are still needed. The significant elevation of plasma TDP‐43 in the ALS group agrees with one case–control study.^4^ The previous measurement of plasma TDP‐43 based on conventional immunoassay failed to accurately quantify more than 70% of samples due to insufficient sensitivity.^4^, ^25^ In contrast, we could detect measurable signals from the whole plasma samples. This performance may be because of the advantage of the Simoa analyzer, with 100‐ to 1000‐fold higher sensitivity than conventional assays^26^. This result provides evidence supporting the potential diagnostic value of plasma TDP‐43 for ALS as well as the usefulness of such new digital analytical platforms for the development of blood–based biomarkers of the disease. Regarding CSF levels of t‐tau in ALS patients, previous studies have yielded similar inconsistent results, which ranged between normal^7^, ^8^, ^15^, ^27^, ^28^ and increased levels.^5^, ^6^, ^29^ This inconsistency might be linked to the inherent variability of the disease; for example, variability in release of tau from motor neurons during disease progression.

The combined analysis leads to a technical problem regarding the interassay concordance of the kits. The correction coefficients were markedly more variable than those expected based on the supplier’s information, possibly attributable to the fact that we used different lot of kits. Such lot‐to‐lot quality variation must be improved to ensure greater reliability of future measurements.

In survival analysis all of the biomarkers except for plasma and CSF NfL failed to exhibit any prognostic value consistently across the discovery and validation cohorts. We previously reported that lower CSF TDP‐43 levels were correlated with shorter survival.^10^. However, this study did not reproduce the results in the discovery and validation cohorts, or on combined analysis. A recent study argued that higher levels of CSF t‐tau are associated with shorter survival.^5^ This result was consistent with that of our discovery cohort, but was not reproduced in either the validation cohort or on combined analysis. This inconsistency may have been caused by the shortness of the follow‐up period in the validation cohort, which was around half of that in the previous study.

We acknowledge that the relatively small sample size was a major limitation of the study. Furthermore, the short follow‐up period may have weakened the statistical power to detect an association between survival and the biomarkers.

In conclusion, NfL levels in CSF and plasma were significantly elevated in the ALS patients compared with controls. Moreover, higher levels of those markers were associated with shorter survival. Both may serve as not only diagnostic biomarkers but also as measures of disease progression. TDP‐43 levels in CSF, which were increased in the ALS patients compared with controls but were not associated with survival periods, may only be useful as a diagnostic biomarker. The discrimination ability between ALS and controls was improved by the combined use of CSF TDP‐43 and CSF NfL, therefore CSF TDP‐43 could serve as a biomarker complementary to NfL in the diagnosis of ALS. Plasma TDP‐43 and CSF t‐tau may be elevated in ALS patients and, therefore, be of diagnostic value; however, the results of this study still need future validation in a larger cohort.

## Author Contributions

T. O. and Y.K assisted with patient enrollment, data analysis, and interpretation. H.T., F.K‐M., and M.S. performed laboratory work and data analysis. Y.T. and Y.N. contributed to data collection. D.A. and T.M. participated in the review and revision of the manuscript. T.K. and T.T were involved with the conceptualization and design of the study, patient enrollment, data collection, interpretation of the data, and review of the manuscript. All authors read and approved the final version of the manuscript.

## Conflict of Interest

The authors have no competing financial interests. Also, no nonfinancial conflicts of interest exist.

## Supporting information


**Figure S1**. ROC analyses of the discovery cohort.
**Figure S2**. Scatter plots of levels of TDP‐43, NfL, and t‐tau in plasma and CSF.
**Figure S3**. ROC analyses of the validation cohort.
**Figure S4**. Scatter plots of biomarker levels in individuals aged no younger than 60 in the validation cohort.
**Figure S5**. Receiver operating characteristic (ROC) analyses for the optimal composite parameters of the discovery and validation cohorts.

**Data S1**. Sample collection and preparation, measurement of NfL, TDP‐43, and t‐tau, and statistics.
**Table S1**. Clinical information and concentrations of biomarkers in the control and ALS groups of the discovery cohort.
**Table S2**. Clinical information and concentrations of biomarkers in the control and ALS groups of the validation cohort.
**Table S3**. Measurements of the internal controls and interassay concordance.
