# Supplementary material for: Combined use of CSF NfL and CSF TDP‐43 improves diagnostic performance in ALS
Source: Ann Clin Transl Neurol. 2019 Nov 19;6(12):2489–502. doi: 10.1002/acn3.50943 (PMC6917342; doi:10.1002/acn3.50943)
Supplement: Supplementary file 1 — Figure S1. ROC analyses of the discovery cohort. Figure S2. Scatter plots of levels of TDP‐43, NfL, and t‐tau in plasma and CSF. Figure S3. ROC analyses of the validation cohort. Figure S4. Scatter plots of biomarker levels in individuals aged no younger than 60 in the validation cohort. Figure S5. Receiver operating characteristic (ROC) analyses for the optimal composite parameters of the discovery and validation cohorts. [file ACN3-6-2489-s001.pdf]

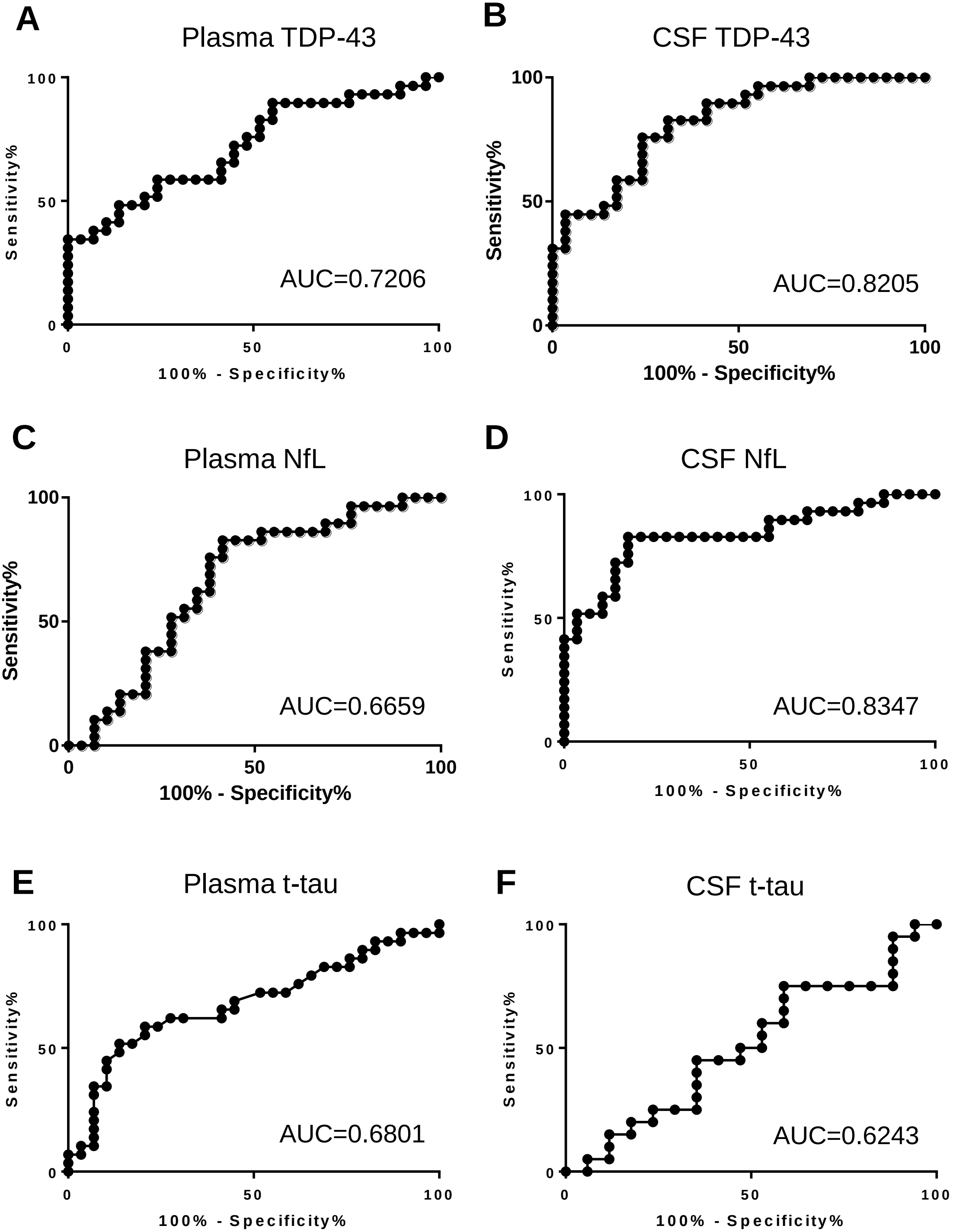

Supplementary Figure 1

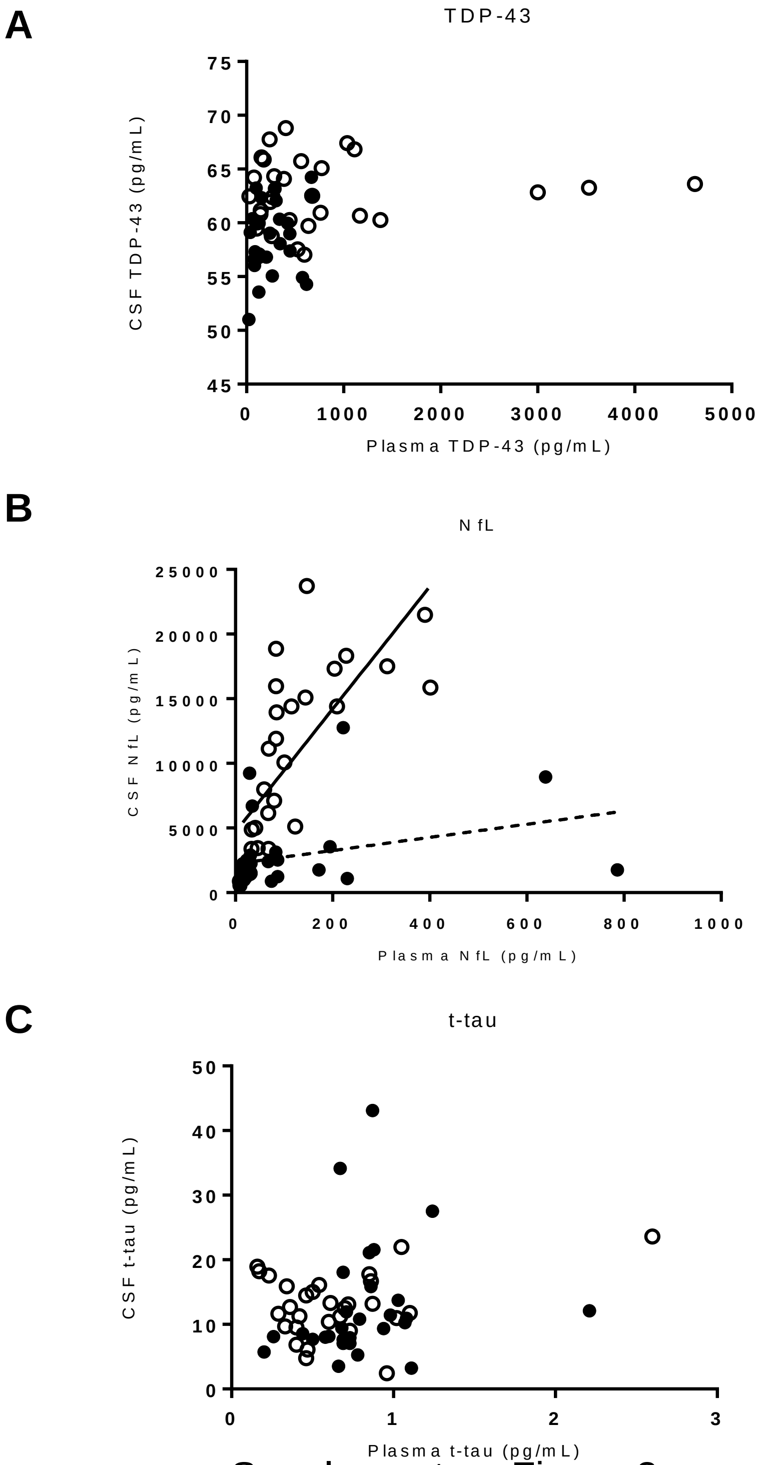

Supplementary Figure 2

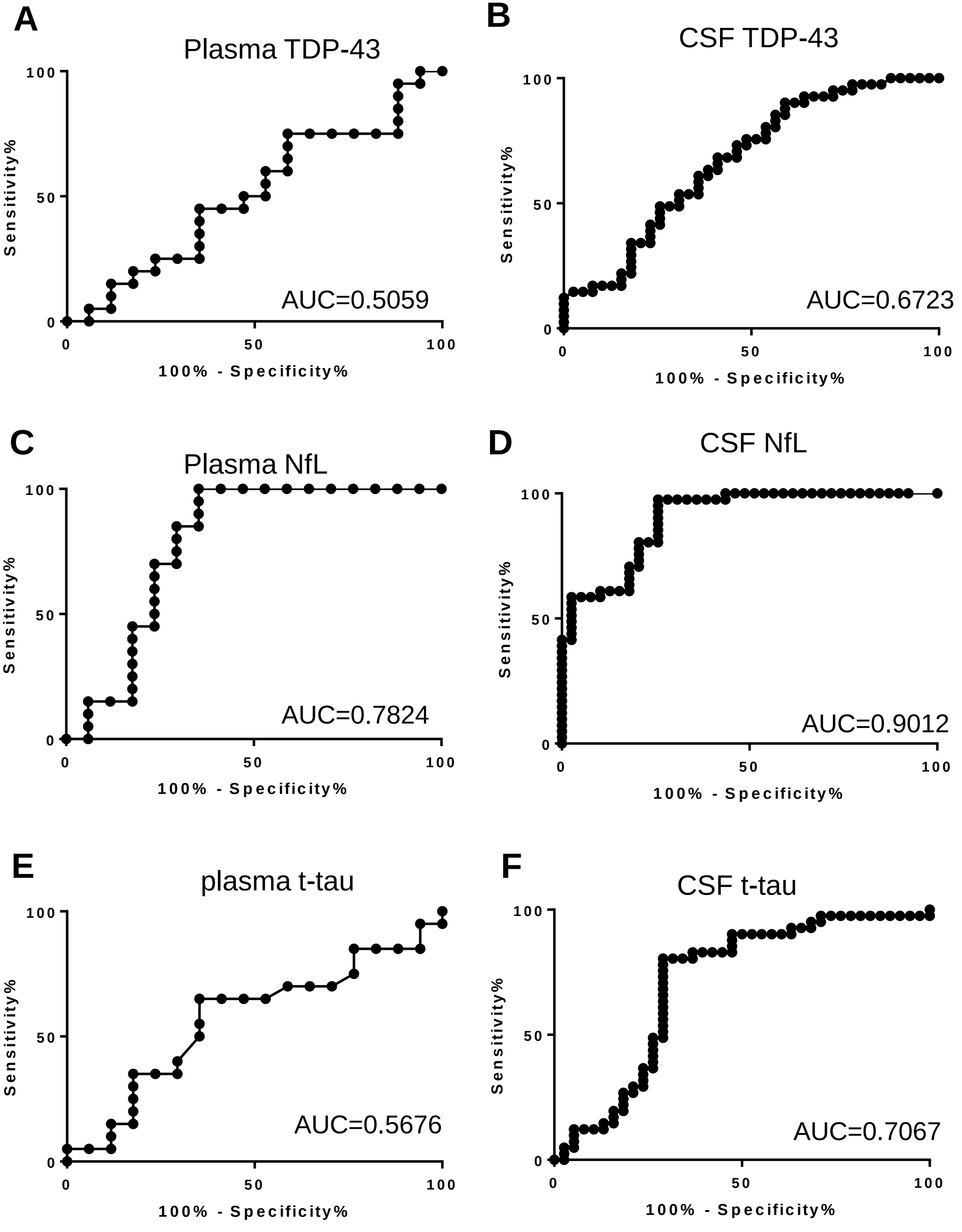

Supplementary Figure 3

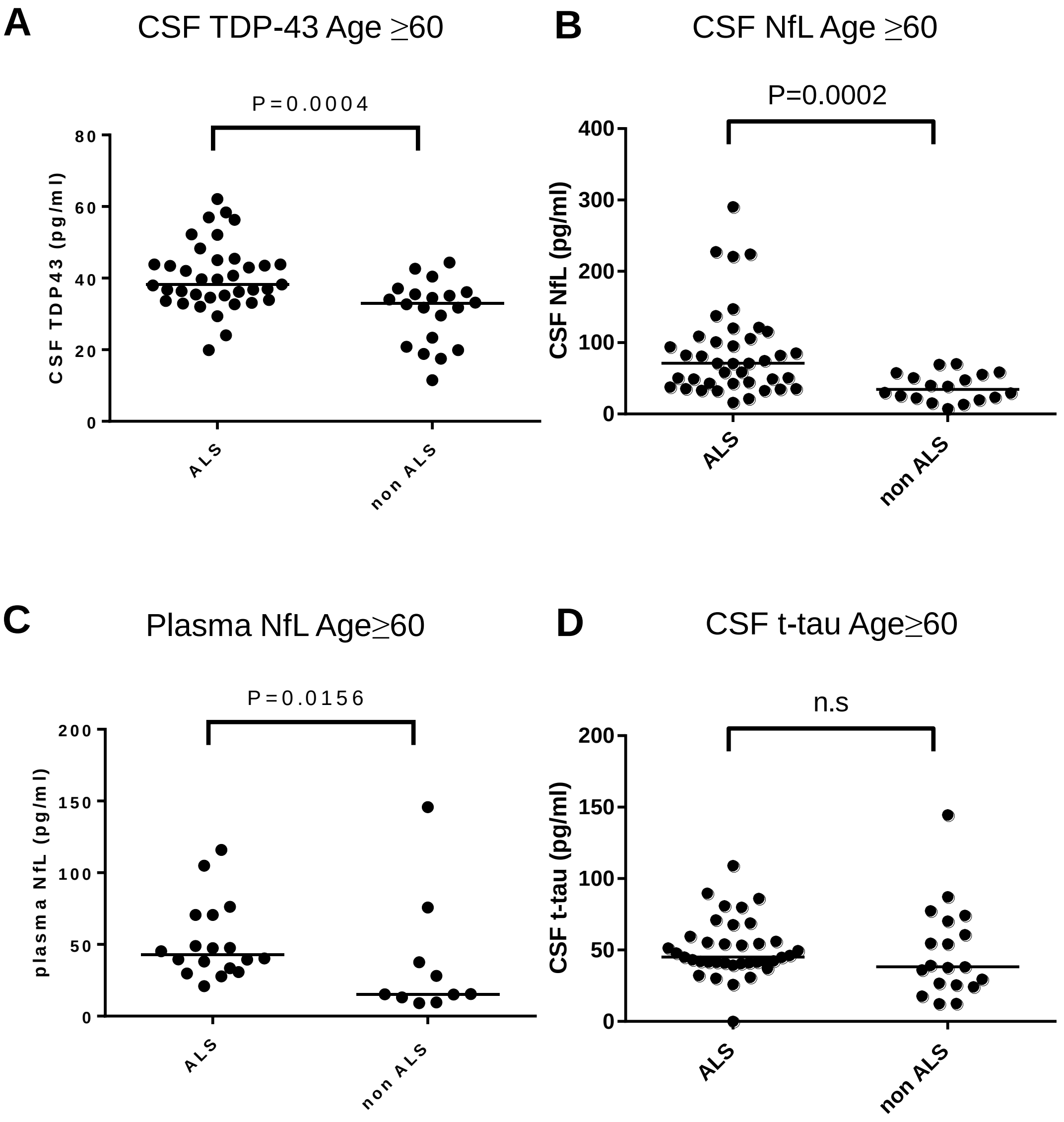

Supplementary Figure 4

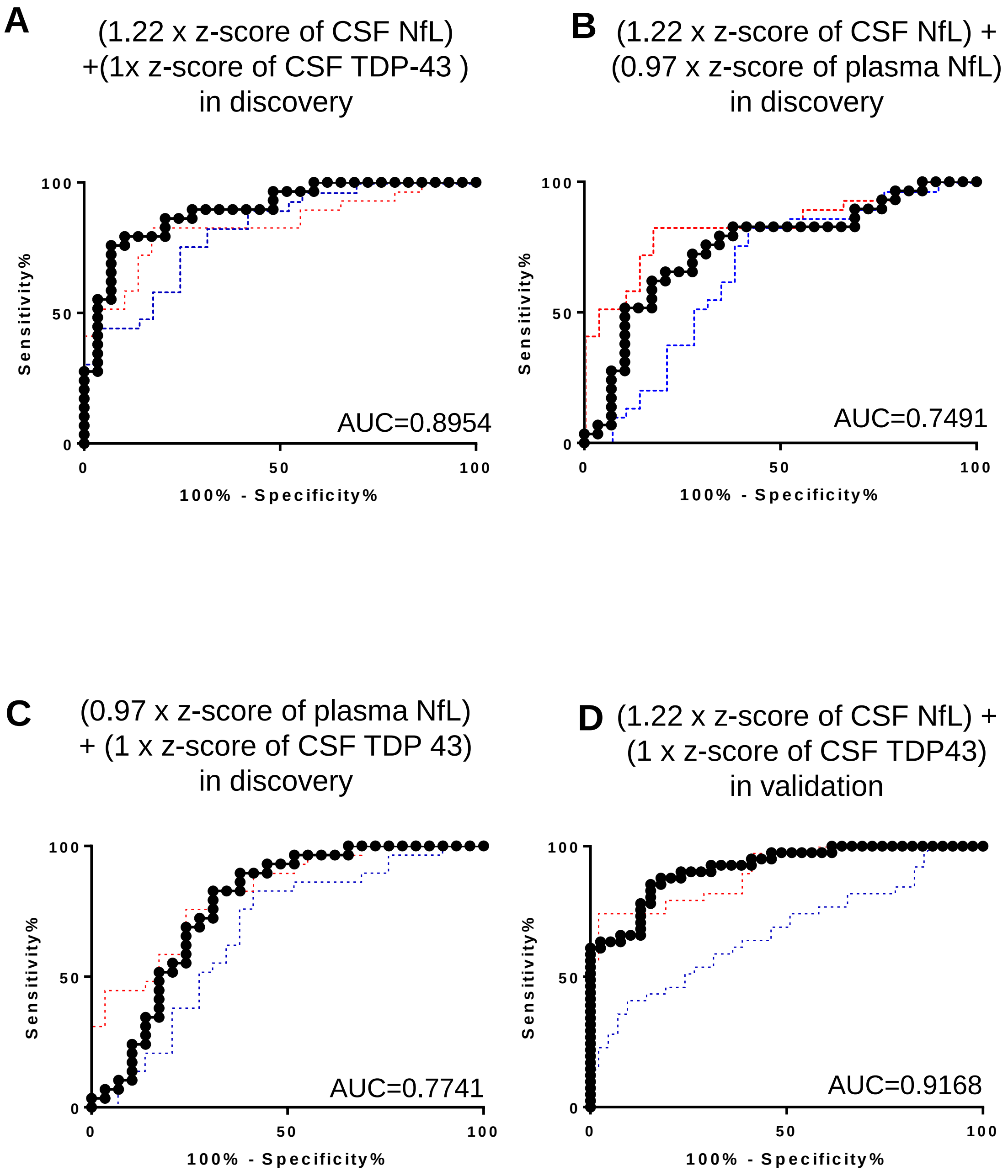

Supplementary Figure 5
