# Supplementary material for: Combined use of CSF NfL and CSF TDP‐43 improves diagnostic performance in ALS
Source: Ann Clin Transl Neurol. 2019 Nov 19;6(12):2489–502. doi: 10.1002/acn3.50943 (PMC6917342; doi:10.1002/acn3.50943)
Supplement: Supplementary file 2 — Data S1. Sample collection and preparation, measurement of NfL, TDP‐43, and t‐tau, and statistics. Table S1. Clinical information and concentrations of biomarkers in the control and ALS groups of the discovery cohort. Table S2. Clinical information and concentrations of biomarkers in the control and ALS groups of the validation cohort. Table S3. Measurements of the internal controls and interassay concordance. [file ACN3-6-2489-s002.docx]

**Supplementary data of Combined use of CSF NfL and CSF TDP-43 improves diagnostic performance in ALS.**

1. Takashi Kasai^1*^, M.D., Ph.D. ([kasaita@koto.kpu-m.ac.jp](mailto:kasaita@koto.kpu-m.ac.jp))
2. Yuta Kojima^1*^, M.D. (ytkjm@koto.kpu-m.ac.jp)
3. Takuma Ohmichi^1^, M.D., Ph.D. (t-omichi@koto.kpu-m.ac.jp)
4. Harutsugu Tatebe^2^, Ph.D. ([tatebe@koto.kpu-m.ac.jp](mailto:tatebe@koto.kpu-m.ac.jp))
5. Yukiko Tsuji^1^, M.D., Ph.D. (y-tsuji@koto.kpu-m.ac.jp)
6. Yu-ichi Noto^1^, M.D., Ph.D. (y-noto@koto.kpu-m.ac.jp)
7. Fukiko Kitani-Morii^1^, M.D., Ph.D. (f-morii@koto.kpu-m.ac.jp)
8. Makiko Shinomoto^1^, M.D. (makiko-t@koto.kpu-m.ac.jp)
9. David Allsop^3^, Ph.D. (d.allsop@lancaster.ac.uk)
10. Toshiki Mizuno^1^, M.D., Ph.D. (mizuno@koto.kpu-m.ac.jp)
11. Takahiko Tokuda^1.4^, M.D., Ph.D. (ttokuda@koto.kpu-m.ac.jp)

^1^ Department of Neurology, Kyoto Prefectural University of Medicine, Kyoto 602-0841, Japan

^2^ Deprtment of Medical Innovation and Translational Medical Science, Kyoto Prefectural University of Medicine, Kyoto 602-0841, Japan

^3^ Division of Biomedical and Life Sciences, Faculty of Health and Medicine, Lancaster University, Lancaster LA1 4YQ, UK

^4^ Department of Molecular Pathobiology of Brain Diseases, Kyoto Prefectural University of Medicine, Kyoto 602-0841, Japan

^*^ These authors equally contributed to this work.

Takashi Kasai completed statistical analysis and list affiliations.

Corresponding authors: Takashi Kasai, M.D., Ph.D. and Takahiko Tokuda, M.D., Ph.D.

Department of Neurology, Kyoto Prefectural University of Medicine, Kyoto 602-0841, Japan. Tel.: +81-75-251-5793 Fax: +81-75-211-8645 E-mail: [kasaita@koto.kpu-m.ac.jp](mailto:kasaita@koto.kpu-m.ac.jp)

**Abstract:** words. **Article body**: words.

**Supplementary methods**

***Diagnostic criteria for neuromuscular diseases***

Patients with neuromuscular diseases are diagnosed according to the following criteria:

Chronic inflammatory demyelinating polyneuropathy (CIDP): definite or probable CIDP in the diagnostic criteria developed by the European Federation of Neurological Societies/Peripheral Nerve Society guideline.^1^

Gullian-Barre syndrome (GBS): level 1 in the Brighton criteria for ^2^

Multifocal motor neuropathy (MMN): definite or probable MMN in the diagnostic criteria developed by the European Federation of Neurological Societies/Peripheral Nerve Society guideline.^3^

Inclusion body myositis (IBM): definite or probable IBM in the diagnostic criteria developed by Needham et al.^4^

***Sample collection and preparation***

Plasma samples were obtained via venous puncture: a total of 8 mL of blood was collected in EDTA-containing tubes. After collection, plasma was separated by centrifugation for 10 min at 3,000 rpm and placed in polypropylene vials. CSF samples were collected in polypropylene vials from cases in the morning (from 9 to 12 a.m.) through lumbar puncture at the L3/L4 or L4/L5 interspace. Immediately after collection, the samples were cleared by centrifugation at 400 x g for 10 min at 4°C. CSF and plasma samples obtained from the enrolled subjects were immediately stored at -80°C until analysis.

***Measurement of NfL, TDP-43, and t-tau***

The plasma and CSF NfL, TDP-43, and t-tau concentrations were measured with Simoa NF-light Advantage Kit, TDP43 Discovery Kit, and Tau 2.0 Advantage Kit, respectively, on a Simoa HD-1 Analyzer (Quanterix, Lexington, MA, USA) according to the protocol issued by the manufacturer. To note, TDP43 Discovery Kit has been developed with a full-length protein calibrator and antibodies against the amino acid residues between 203 – 209 and the C-terminal region of TDP-43. All samples were analyzed in duplicate on one occasion. All samples from cases and controls were evenly distributed on examination.

***Statistics***

The level of significance was set at P<0.05. A comparison between the two independent groups was performed using unpaired Mann-Whitney’s U test. Multiple comparisons were tested with the Kruskal-Wallis test and Dunn’s multiple comparison procedure. The Chi-square test was used to evaluate the significance of categorical variables. Correlation analysis was conducted using Spearman’s rank correlation coefficient test. Receiver operating characteristic curve (ROC) analysis was used to determine the best cut-off values for the biomarkers. Survival curves were estimated by the Kaplan-Meier method and differences in survival were measured by the log-rank test. Analyses were performed using GraphPad Prism version 6 software (GraphPad Software, San Diego, USA). Multivariate analyses of the risk for endpoints associated with selected independent variables were performed using a Cox proportional hazard model. Primary component analysis was conducted to generate optimal weighting for z-scores of biomarkers. Those analyses were performed using SPSS for Windows version 23 software (IBM Japan Ltd., Tokyo, Japan).

**Supplementary Table 1A** Clinical information and concentrations of biomarkers in the control group of the discovery cohort.

| Case | Sex | Age (years) | Comorbid disease or condition | Plasma  TDP-43  (pg/mL) | CSF  TDP-43  (pg/mL) | Plasma  NfL  (pg/mL) | CSF  NfL  (pg/mL) | Plasma  t-tau  (pg/mL) | CSF  t-tau  (pg/mL) |
| --- | --- | --- | --- | --- | --- | --- | --- | --- | --- |
| 1 | F | 68 | Neuropathy | 23.88 | 51.01 | 21.39 | 1,199.42 | 0.98 | 11.42 |
| 2 | F | 71 | GBS | 346.15 | 58.06 | 87.06 | 1,235.28 | 0.20 | 5.73 |
| 3 | F | 64 | Headache | 204.85 | 56.82 | 34.59 | 6,700.98 | 0.66 | 3.52 |
| 4 | M | 61 | GBS | 617.91 | 54.30 | 29.15 | 2,165.14 | 0.44 | 8.58 |
| 5 | M | 67 | Lumbar radiculopathy | 126.14 | 57.12 | 7.47 | 542.75 | 1.11 | 3.22 |
| 6 | M | 76 | Neuropathy | 445.14 | 58.99 | 8.46 | 878.31 | 0.26 | 8.09 |
| 7 | M | 45 | Anxiety neurosis | 78.29 | 56.61 | 87.03 | 2,534.21 | 0.60 | 8.15 |
| 8 | M | 46 | Myopathy | 59.01 | 60.39 | 230.00 | 1,098.62 | 0.50 | 7.70 |
| 9 | M | 68 | MFS | 423.46 | 59.95 | 32.11 | 2,266.05 | 1.08 | 10.94 |
| 10 | M | 77 | Neuropathy | 265.00 | 55.06 | 17.88 | 2,054.75 | 0.69 | 7.06 |
| 11 | M | 75 | Cervical radiculopathy | 81.50 | 56.05 | 83.11 | 3,131.24 | 1.24 | 27.51 |
| 12 | M | 64 | Normal | 124.09 | 56.76 | 31.78 | 1,479.34 | 0.69 | 18.05 |
| 13 | F | 75 | Epilepsy | 126.50 | 53.57 | 11.36 | 1,068.63 | 0.85 | 21.11 |
| 14 | M | 67 | Neuropathy | 662.39 | 62.56 | 29.18 | 9,242.09 | 0.86 | 15.85 |
| 15 | F | 66 | Spastic paraparesis | 136.09 | 57.07 | 7.42 | 574.07 | 0.94 | 9.37 |
| 16 | M | 69 | Cervical radiculopathy | 448.77 | 57.39 | 786.28 | 1,757.95 | 0.69 | 7.66 |
| 17 | F | 47 | Anxiety neurosis | 88.76 | 57.32 | 194.49 | 3,545.12 | 1.07 | 10.25 |
| 18 | F | 65 | MFS | 293.19 | 63.17 | 67.71 | 2,410.12 | 0.71 | 11.95 |
| 19 | F | 72 | CIDP | 154.17 | 62.35 | 74.36 | 881.87 | 0.78 | 5.28 |
| 20 | M | 50 | Neuropathy | 287.83 | 63.19 | 9.87 | 1,008.40 | 0.68 | 9.42 |
| 21 | M | 61 | CIDP | 576.48 | 54.92 | 23.09 | 2,509.79 | 0.73 | 7.08 |
| 22 | F | 68 | Cervical radiculopathy | 37.47 | 59.11 | 20.81 | 1,357.05 | 2.21 | 12.12 |
| 23 | M | 63 | Epilepsy | 669.21 | 64.24 | 222.07 | 12,763.62 | 0.88 | 21.57 |
| 24 | M | 70 | Cranial neuropathy | 239.12 | 59.03 | 31.05 | 2,902.72 | 0.73 | 7.92 |
| 25 | M | 68 | Epilepsy | 339.83 | 60.35 | 19.55 | 1,513.47 | 0.87 | 43.10 |
| 26 | F | 71 | None | 128.27 | 59.97 | 15.33 | 2,239.24 | 1.03 | 13.74 |
| 27 | M | 67 | Cervical radiculopathy | 110.63 | 59.90 | 638.53 | 8,947.07 | 0.67 | 34.13 |
| 28 | M | 83 | Neuropathy | 97.86 | 63.23 | 172.16 | 1,759.59 | 0.79 | 10.80 |
| 29 | M | 74 | GBS | 303.14 | 62.06 | 10.93 | 536.04 | 0.58 | 8.01 |
|  | M:F  19:10 | Mean±SD  66.1±9.2 |  | Mean±SD  258.45  ±194.74 | Mean±SD  58.63  ±3.28 | Mean±SD  103.59  ±181.80 | Mean±SD  2769.07  ±2931.36 | Mean±SD  0.81  ±0.36 | Mean±SD  12.74  ±9.11 |

GBS: Gullain-Barre syndrome, MFS: Millar-Fisher syndrome, CIDP: chronic inflammatory demyelinating polyneuropathy, MMN: multifocal motor neuropathy

**Supplementary Table 1B** Clinical information and concentrations of biomarkers in the ALS group of the discovery cohort.

| Case | Sex | Age (years) | Diagnosis | Disease duration (Months) | Bullbar onset | Dementia | Follow-up period (days) | Plasma  TDP-43  (pg/mL) | CSF  TDP-43  (pg/mL) | Plasma  NfL  (pg/mL) | CSF  NfL  (pg/mL) | Plasma  t-tau  (pg/mL) | CSF  t-tau  (pg/mL) |
| --- | --- | --- | --- | --- | --- | --- | --- | --- | --- | --- | --- | --- | --- |
| 1 | F | 59 | Definite | 46 | + | - | 734 | 236.99 | 67.77 | 40.58 | 5,017.91 | 0.67 | 11.24 |
| 2 | M | 75 | Probable | 8 | + | + | 17 | 77.59 | 64.22 | 209.40 | 1,4408.29 | 0.72 | 13.11 |
| 3 | F | 60 | Probable | 11 | - | - | 2,483 | 283.77 | 64.33 | 33.84 | 4,883.56 | 0.50 | 15.01 |
| 4 | M | 73 | Probable | 10 | + | - | 2,793 | 145.63 | 61.17 | 19.65 | 1,263.61 | 0.46 | 4.76 |
| 5 | M | 65 | Possible | 11 | - | - | 575 | 268.25 | 62.39 | 83.46 | 15,970.56 | 1.10 | 11.79 |
| 6 | M | 57 | Definite | 14 | + | + | 45 | 27.87 | 62.45 | 68.36 | 11,126.48 | 0.47 | 6.09 |
| 7 | M | 79 | Definite | 128 | + | - | 265 | 103.36 | 59.45 | 122.89 | 5,116.48 | 0.36 | 12.69 |
| 8 | M | 65 | Probable | 32 | - | - | 645 | 561.70 | 65.74 | 100.62 | 10,081.05 | 0.17 | 18.21 |
| 9 | M | 56 | Possible | 6 | - | - | 1,534 | 1,380.04 | 60.26 | 15.67 | 1,052.22 | 0.40 | 6.84 |
| 10 | F | 62 | Probable | 10 | - | - | 2,590 | 636.16 | 59.70 | 17.69 | 1,705.62 | 0.29 | 11.64 |
| 11 | F | 74 | Possible | 8 | + | + | 1,313 | 243.16 | 61.94 | 79.48 | 7,122.37 | 0.85 | 17.77 |
| 12 | M | 62 | Probable | 5 | - | - | 548 | 1,166.76 | 60.65 | 83.58 | 18,872.17 | 0.34 | 15.89 |
| 13 | F | 72 | Probable | 13 | + | - | 219 | 773.75 | 65.10 | 312.68 | 17,505.79 | 0.86 | 16.68 |
| 14 | M | 63 | Probable | 11 | + | + | 355 | 259.70 | 58.77 | 204.25 | 17,319.04 | 0.87 | 13.19 |
| 15 | F | 85 | Possible | 31 | + | + | 2,257 | 595.43 | 57.03 | 46.09 | 3,454.52 | 1.05 | 21.96 |
| 16 | M | 74 | Probable | 7 | - | - | 353 | 384.02 | 64.08 | 144.16 | 15,092.26 | 2.60 | 23.62 |
| 17 | M | 38 | Probable | 33 | - | - | 40 | 1,037.41 | 67.41 | 8.93 | 878.75 | 0.33 | 9.69 |
| 18 | M | 81 | Probable | 9 | - | - | 293 | 525.45 | 57.54 | 228.15 | 18,322.21 | 0.54 | 16.10 |
| 19 | F | 43 | Probable | 8 | + | - | 2,274 | 142.64 | 60.82 | 115.04 | 1,4403.38 | 0.73 | 9.04 |
| 20 | F | 71 | Possible | 18 | - | - | 1,008 | 762.50 | 60.94 | 68.25 | 3,386.35 | 0.16 | 18.93 |
| 21 | M | 75 | Definite | 10 | + | + | 122 | 1,113.44 | 66.85 | 401.30 | 15,855.66 | 0.42 | 11.25 |
| 22 | F | 59 | Definite | 56 | - | + | 597 | 154.32 | 66.08 | 33.32 | 3,376.32 | 0.23 | 17.56 |
| 23 | M | 72 | Possible | 57 | - | - | 2,100 | 175.78 | 65.88 | 29.24 | 1,519.83 | 0.96 | 2.45 |
| 24 | M | 53 | Possible | 10 | - | - | 1,764 | 4,619.90 | 63.62 | 83.56 | 11,899.70 | 1.02 | 10.99 |
| 25 | F | 76 | Definite | 11 | + | - | 346 | 3,527.99 | 63.25 | 67.48 | 6,148.49 | 0.46 | 14.49 |
| 26 | M | 60 | Definite | 39 | + | - | 613 | 3,000.11 | 62.83 | 84.59 | 13,941.78 | 0.40 | 9.53 |
| 27 | F | 66 | Possible | 7 | - | - | 80 | 675.93 | 62.52 | 147.13 | 23,720.46 | 0.61 | 13.33 |
| 28 | M | 38 | Possible | 7 | - | - | 277 | 441.77 | 60.25 | 59.17 | 7,983.78 | 0.60 | 10.39 |
| 29 | M | 84 | Definite | 5 | + | + | 100 | 403.74 | 68.80 | 390.33 | 21,485.16 | 0.70 | 12.52 |
|  | M:F  18:11 | Mean±SD  65.41  ±12.34 | Definite: N=8  Probable: N=12  Possible: N=9 | Median, (range)  =11  (5-128) | Bullbar onset  N=14 | Dementia  N=8 | Median, (range)=575,  (17-2,793) | Mean±SD  818.11  ±1,084.06 | Mean±SD  62.82  ±3.08 | Mean±SD  113.75  ±105.91 | Mean±SD  10,100.48  ±6,932.43 | Mean±SD  0.65  ±0.46 | Mean±SD  12.99  ±4.86 |

Nineteen patients who died, received tracheostomy, or needed invasive ventilation during the follow-up period are indicated by gray shading.

**Supplementary Table 2A** Clinical information and concentrations of biomarkers in the control group of the validation cohort.

| Case | Sex | Age (years) | Comorbid disease or condition | Plasma  TDP-43  (pg/mL) | CSF  TDP-43  (pg/mL) | Plasma  NfL  (pg/mL) | CSF  NfL  (pg/mL) | Plasma  t-tau  (pg/mL) | CSF  t-tau  (pg/mL) |
| --- | --- | --- | --- | --- | --- | --- | --- | --- | --- |
| 1 | F | 58 | CIDP | N/A | 51.88 | N/A | 1,167.73 | N/A | 29.93 |
| 2 | M | 60 | CIDP | N/A | 35.47 | N/A | 1,342.37 | N/A | 37.72 |
| 3 | M | 35 | CIDP | N/A | 15.08 | N/A | 1,077.80 | N/A | 27.29 |
| 4 | M | 56 | CIDP | N/A | 31.02 | N/A | 1,801.90 | N/A | 15.70 |
| 5 | F | 80 | CIDP | N/A | 18.84 | N/A | 3,014.18 | N/A | 38.18 |
| 6 | M | 65 | CIDP | N/A | 40.45 | N/A | 2,556.27 | N/A | 29.66 |
| 7 | M | 59 | CIDP | N/A | 22.85 | N/A | 1,101.44 | N/A | 24.66 |
| 8 | M | 38 | CIDP | 70.19 | 39.35 | 2.48 | 1,210.84 | 0.36 | 44.64 |
| 9 | M | 39 | CIDP | N/A | 37.80 | N/A | 858.99 | N/A | 33.17 |
| 10 | M | 72 | CIDP | 363.95 | 37.12 | 15.34 | 1,539.55 | 0.32 | 26.73 |
| 11 | M | 67 | CIDP | N/A | 20.84 | N/A | 6,958.25 | N/A | 54.30 |
| 12 | F | 73 | CIDP | N/A | 36.12 | N/A | 762.31 | N/A | 17.82 |
| 13 | M | 60 | CIDP | N/A | 19.90 | N/A | 2,943.78 | N/A | 12.65 |
| 14 | M | 45 | CIDP | 461.28 | 25.85 | 47.05 | 1,439.19 | 2.10 | 19.89 |
| 15 | M | 65 | CIDP | 104.64 | 44.36 | 75.81 | 5,534.06 | 0.29 | 24.17 |
| 16 | M | 62 | CIDP | 255.94 | N/A | 9.15 | N/A | 0.49 | N/A |
| 17 | M | 87 | CIDP | 97.92 | 23.34 | 28.09 | 7,038.53 | 0.47 | 70.22 |
| 18 | M | 31 | GBS | N/A | 31.23 | N/A | 858.82 | N/A | 27.78 |
| 19 | F | 31 | GBS | N/A | 45.88 | N/A | 1,411.65 | N/A | 23.86 |
| 20 | M | 38 | GBS | N/A | 47.69 | N/A | 3,183.92 | N/A | 12.96 |
| 21 | F | 71 | GBS | N/A | 42.66 | N/A | 2,336.52 | N/A | 60.72 |
| 22 | M | 93 | GBS | N/A | 11.52 | N/A | 0.00 | N/A | 144.61 |
| 23 | F | 79 | GBS | N/A | 34.06 | N/A | 4,009.68 | N/A | 74.23 |
| 24 | M | 59 | GBS | N/A | 50.06 | N/A | 0.00 | N/A | 75.00 |
| 25 | M | 43 | GBS | N/A | 29.21 | N/A | 1,194.88 | N/A | 50.29 |
| 26 | M | 74 | GBS | N/A | 34.47 | N/A | 2,244.64 | N/A | 54.75 |
| 27 | M | 46 | GBS | N/A | 38.87 | N/A | 687.38 | N/A | 22.68 |
| 28 | F | 28 | GBS | N/A | 47.56 | N/A | 1,891.14 | N/A | 34.46 |
| 29 | F | 36 | GBS | N/A | 42.58 | N/A | 664.86 | N/A | 25.23 |
| 30 | F | 23 | GBS | N/A | 9.05 | N/A | 1,480.22 | N/A | 2.65 |
| 31 | M | 22 | GBS | 1024.60 | 52.13 | 5.58 | 589.87 | 0.09 | 12.18 |
| 32 | F | 95 | GBS | 151.23 | 17.53 | 37.57 | 5,086.93 | 1.72 | 87.20 |
| 33 | M | 29 | GBS | 183.48 | N/A | 73.08 | N/A | 0.25 | N/A |
| 34 | M | 66 | GBS | 283.64 | 31.78 | 145.72 | 3,899.03 | 0.35 | 12.49 |
| 35 | M | 48 | GBS | 30.18 | N/A | 3.87 | N/A | 0.39 | N/A |
| 36 | M | 63 | MMN | N/A | 29.56 | N/A | 4,794.38 | N/A | 25.65 |
| 37 | M | 33 | MMN | 99.56 | 36.57 | 3.36 | 8,327.17 | 0.70 | 33.51 |
| 38 | M | 68 | MMN | N/A | 33.18 | N/A | 1,982.28 | N/A | 39.22 |
| 39 | M | 69 | MMN | 286.26 | 32.73 | 13.00 | 5,894.34 | 0.13 | 36.08 |
| 40 | F | 19 | MMN | N/A | 36.68 | N/A | 2,532.55 | N/A | 28.02 |
| 41 | M | 39 | MMN | 198.15 | N/A | 0.00 | N/A | 0.18 | N/A |
| 42 | M | 79 | IBM | N/A | 31.74 | N/A | 5,790.89 | N/A | 77.49 |
| 43 | F | 78 | IBM | 138.06 | N/A | 9.61 | N/A | 0.53 | N/A |
| 44 | M | 75 | IBM | 65.31 | N/A | 15.01 | N/A | 0.19 | N/A |
| 45 | M | 75 | IBM | 282.18 | N/A | 15.23 | N/A | 0.28 | N/A |
| 46 | M | 73 | IBM | N/A | 35.07 | N/A | 0.00 | N/A | N/A |
|  | M:F  34:12 | Mean±SD  69.83  ±20.18 | CIDP: N=17  GBS: N=18  MMN: N=6  IBM: N=5 | Mean±SD  170.93  ±233.29  N=17 | Mean±SD  33.40  ±10.92  N=39 | Mean±SD  9.96  ±38.03  N=17 | Mean±SD  28.95.45  ±2,154.83  N=39 | Mean±SD  0.29  ±0.55  N=17 | Mean±SD  77.49±27.08  N=39 |

CIDP: chronic inflammatory demyelinating polyneuropathy, GBS: Gullain-Barre syndrome, MMN: multifocal motor neuropathy, IBM: inclusion body myositis, N/A: not available.

Supplementary **Table 2B** Clinical information and concentrations of biomarkers in the ALS group of the validation cohort.

| Case | Sex | Age (years) | Diagnosis | Disease duration (Months) | Bullbar onset | Dementia | Follow-up period (days) | Plasma  TDP-43  (pg/mL) | CSF  TDP-43  (pg/mL) | Plasma  NfL  (pg/mL) | CSF  NfL  (pg/mL) | Plasma  t-tau  (pg/mL) | CSF  t-tau  (pg/mL) |
| --- | --- | --- | --- | --- | --- | --- | --- | --- | --- | --- | --- | --- | --- |
| 1 | F | 75 | Definite | 5 | - | - | 189 | N/A | 24.06 | N/A | 2,9043.55 | N/A | 89.71 |
| 2 | M | 79 | Definite | 16 | - | - | 835 | N/A | 32.68 | N/A | 3,488.77 | N/A | 56.03 |
| 3 | M | 56 | Suspected | 6 | - | - | 789 | N/A | 31.63 | N/A | 15,000.85 | N/A | 24.48 |
| 4 | F | 65 | Suspected | 18 | - | - | 951 | N/A | 52.22 | N/A | 3,274.41 | N/A | 79.95 |
| 5 | F | 65 | Possible | 14 | - | - | 971 | N/A | 43.42 | N/A | 3,784.68 | N/A | 41.36 |
| 6 | F | 81 | Probable | 13 | + | - | 491 | N/A | 39.64 | N/A | 4,916.65 | N/A | 41.02 |
| 7 | M | 51 | Possible | 41 | + | - | 1592 | N/A | 42.02 | N/A | 4,390.66 | N/A | 43.05 |
| 8 | M | 78 | Probable | 8 | - | - | 94 | N/A | 33.87 | N/A | 3,533.72 | N/A | 46.33 |
| 9 | M | 74 | Possible | 68 | - | + | 194 | N/A | 56.98 | N/A | 4,285.75 | N/A | 32.29 |
| 10 | F | 71 | Suspected | 17 | - | - | 1,022 | N/A | 45.03 | N/A | 3,554.32 | N/A | 67.78 |
| 11 | M | 77 | Definite | 11 | - | + | 88 | N/A | 42.03 | N/A | 8,208.41 | N/A | 41.78 |
| 12 | F | 75 | Possible | 3 | + | + | 318 | N/A | 33.13 | N/A | 5,070.06 | N/A | 86.01 |
| 13 | F | 77 | Definite | 14 | + | + | 465 | N/A | 43.84 | N/A | 10,572.57 | N/A | 80.93 |
| 14 | F | 76 | Definite | 15 | - | - | 239 | N/A | 45.42 | N/A | 12,144.97 | N/A | 109.09 |
| 15 | F | 66 | Definite | 7 | + | - | 713 | N/A | 62.11 | N/A | 3,238.51 | N/A | 44.94 |
| 16 | M | 78 | Definite | 44 | + | - | 179 | N/A | 58.35 | N/A | 3,324.41 | N/A | 0.00 |
| 17 | F | 77 | Definite | 9 | - | - | 209 | N/A | 43.47 | N/A | 7,119.34 | N/A | 40.57 |
| 18 | M | 69 | Possible | 20 | - | - | 286 | N/A | 19.90 | N/A | 8,104.97 | N/A | 39.34 |
| 19 | F | 66 | Possible | 7 | - | - | 74 | 391.04 | 38.23 | 47.37 | 22,404.23 | 0.20 | 59.65 |
| 20 | F | 78 | Possible | 8 | + | - | 524 | N/A | 48.31 | N/A | 4,919.83 | N/A | 54.55 |
| 21 | M | 73 | Definite | 7 | - | + | 28 | N/A | 52.13 | N/A | 2,146.78 | N/A | 42.35 |
| 22 | M | 37 | Probable | 7 | - | - | 271 | 760.39 | 36.59 | 20.20 | 11,514.47 | 0.47 | 40.29 |
| 23 | M | 77 | Probable | 9 | - | - | 89 | 234.45 | N/A | 39.51 | N/A | 0.32 | N/A |
| 24 | F | 85 | Probable | 18 | + | - | 137 | N/A | 43.83 | N/A | 9,406.32 | N/A | 42.06 |
| 25 | F | 72 | Definite | 31 | - | - | 41 | 235.11 | 40.70 | 33.46 | 7,487.61 | 0.64 | 55.48 |
| 26 | M | 75 | Definite | 6 | + | + | - | 155.49 | N/A | 20.92 | N/A | 0.20 | N/A |
| 27 | M | 84 | Definite | 129 | + | + | 42 | 204.18 | 32.04 | 115.98 | 22,735.61 | 0.46 | 47.97 |
| 28 | M | 73 | Definite | 15 | - | - | 178 | 132.86 | 36.78 | 104.89 | 8,242.14 | 0.89 | 37.02 |
| 29 | M | 68 | Definite | 23 | - | - | 367 | N/A | 56.30 | N/A | 9,554.94 | N/A | 43.24 |
| 30 | F | 67 | Probable | 21 | + | - | 493 | N/A | 34.56 | N/A | 7,118.70 | N/A | 51.47 |
| 31 | M | 76 | Suspected | 15 | - | - | 565 | N/A | 36.96 | N/A | 4,481.33 | N/A | 71.07 |
| 32 | M | 53 | Probable | 12 | + | - | 459 | 66.95 | 40.01 | 55.12 | 10,776.87 | 0.00 | 25.19 |
| 33 | M | 70 | Definite | 25 | - | + | 372 | 194.35 | 32.92 | 76.27 | 12,049.33 | 0.40 | 25.96 |
| 34 | M | 76 | Probable | 22 | + | - | 439 | N/A | 39.72 | N/A | 10,115.29 | N/A | 68.89 |
| 35 | M | 63 | Probable | 22 | - | - | 418 | 428.40 | 36.39 | 39.43 | 10,903.74 | 0.40 | 41.57 |
| 36 | M | 70 | Definite | 13 | - | - | 58 | 68.62 | 36.16 | 45.31 | 7,080.63 | 0.77 | 40.85 |
| 37 | M | 81 | Definite | 12 | - | - | 154 | 378.70 | 29.37 | 70.51 | 22,079.04 | 0.48 | 45.16 |
| 38 | M | 69 | Probable | 6 | - | - | 95 | 91.74 | N/A | 70.50 | N/A | 0.54 | N/A |
| 39 | F | 83 | Probable | 16 | - | - | 306 | 98.66 | 37.96 | 38.15 | 5,061.10 | 0.66 | 41.62 |
| 40 | M | 79 | Suspected | 43 | - | - | 459 | N/A | 42.97 | N/A | 4,330.97 | N/A | 49.64 |
| 41 | M | 62 | Definite | 7 | + | - | 554 | 191.84 | 35.41 | 40.28 | 11,597.32 | 0.25 | 30.25 |
| 42 | M | 76 | Possible | 24 | + | + | 549 | 372.33 | 36.74 | 29.80 | 5,869.80 | 2.57 | 54.19 |
| 43 | M | 71 | Probable | 27 | - | - | 243 | 117.62 | 33.64 | 47.61 | 14,743.79 | 0.11 | 30.90 |
| 44 | M | 78 | Probable | 25 | - | - | 235 | 33.87 | 35.17 | 48.95 | 8,539.40 | 0.47 | 53.48 |
| 45 | F | 60 | Probable | 5 | - | - | 258 | 106.88 | N/A | 30.73 | N/A | 0.67 | N/A |
| 46 | M | 71 | Probable | 31 | - | - | 510 | 128.54 | N/A | 27.67 | N/A | 0.11 | N/A |
|  | M:F  29:17 | Mean±SD  71.36  ±9.27 | Definite: N=18  Probable: N=15  Possible: N=8  Suspected: N=5 | Median, (range)  =15  (3-129) | Bullbar onset  N=15 | Dementia  N=9 | Median, (range)=305 (28-1592) | Mean±SD  158.51  ±173.32  N=20 | Mean±SD  35.24  ±8.88  N=41 | Mean±SD  37.51  ±25.85  N=20 | Mean±SD  10,187.58±6,104.26  N=41 | Mean±SD  0.70  ±0.53  N=20 | Mean±SD  42.21  ±20.08  N=41 |

Twenty-six patients who died, received tracheostomy, or needed invasive ventilation during the follow-up are indicated by gray shading. Case 36 reached the endpoint a few days after sample collection; therefore, we excluded this case from the following survival analysis.

**Supplementary Table 3** Raw data of internal controls

|  | TDP43 plasma | TDP43 CSF | | NfL plasma | NfL CSF | Tau plasma | Tau CSF |
| --- | --- | --- | --- | --- | --- | --- | --- |
| Discovery |  |  |  | |  |  |  |
| Case 1. | 175.78 | 65.88 | 29.24 | | 1519.83 | 0.96 | 2.45 |
| Case 2. | 3527.99 | 63.25 | 67.48 | | 6148.49 | 0.46 | 14.49 |
| Case 3. | 3000.11 | 62.83 | 84.59 | | 13941.78 | 0.40 | 9.53 |
| Case 4. | 403.74 | 68.80 | 390.33 | | 21485.16 | 0.70 | 12.52 |
| Validation |  |  |  | |  |  |  |
| Case 1. | 122.04 | 31.51 | 9.55 | | 1612.15 | 0.52 | 11.87 |
| Case 2. | 1450.97 | 47.70 | 21.88 | | 5834.32 | 0.29 | 50.04 |
| Case 3. | 1758.47 | 45.03 | 27.80 | | 13770.82 | 0.32 | 36.09 |
| Case 4. | 204.18 | 32.04 | 115.98 | | 22735.61 | 0.46 | 47.97 |
| Correction factor | 2.01 | 1.67 | 3.26 | | 0.98 | 1.58 | 0.27 |

Note: The correction factors except for that of NfL CSF were markedly inferior to the inter-assay coefficient variation reported by the supplier. This is considered to be mainly due to lot-to-lot quality variation. We used lots of kits for validation measurement different from those in the discovery study.

**Supplementary figure legends**

**Supplementary Figure 1**

ROC analyses of the discovery cohort.

AUC values are indicated in the graphs. The title of each graph represents the biomarker used as an independent variable on analysis: (A): plasma TDP-43, (B): CSF TDP-43, (C): plasma NfL, (D): CSF NfL, (E): plasma t-tau, and (F): CSF t-tau

**Supplementary Figure 2**

Scatter plots of levels of TDP-43 (A), NfL (B), and t-tau (C) in plasma and CSF are presented. The scales of the X- and Y-axis represent the CSF and plasma concentrations of each biomarker, respectively. Black and white circles indicate individuals in the control and ALS groups, respectively. A significant positive correlation between NfL levels of plasma and CSF in the ALS group of the discovery cohort was observed (solid line: P<0.0001, Spearman’s r=0.8857). Such CSF-plasma correlation was also significant in the control group (dashed line: P=0.0013, Spearman’s r=0.5680). Neither TDP-43 nor t-tau levels showed any plasma-CSF correlation in either of the groups (TDP-43 in the ALS group: P=0.9252, TDP-43 in the control group: P=0.2279, t-tau in the ALS group: P=0.1024, t-tau in the control group: P=0.3463).

**Supplementary Figure 3**

ROC analyses of the validation cohort.

AUC values are indicated in the graphs. The title of each graph represents the biomarker used as an independent variable on analysis: (A): plasma TDP-43, (B): CSF TDP-43, (C): plasma NfL, (D): CSF NfL, (E): plasma t-tau, and (F): CSF t-tau.

**Supplementary Figure 4**

Scatter plots of levels of biomarkers in individuals aged no younger than 60 in the validation cohort. Comparisons between the ALS (n=42) and control (n=24) groups regarding CSF TDP-43 (A), CSF NfL (B), plasma NfL (C), and CSF t-tau (D) are presented. Bars indicate median values. P-values generated by Mann-Whitney’s U test between the ALS and control groups is shown above each graph. n.s: not significant.

**Supplementary Figure 5**

Receiver operating characteristic (ROC) analyses for the optimal composite parameters of the discovery and validation cohorts.

In the main article, we used composite biomarkers calculated by simply multiplying the two biomarkers. This means that the weights of CSF TDP-43, CSF NfL, and plasma NfL were pre-determined as 1: 1. However, there could have been better proportions calculated to generate the composite biomarkers. To test this possibility, we estimated the weight optimal for the discovery dataset using principal component analysis by inputting z-scores of CSF TDP-43, CSF NfL, and plasma NfL. Based on the results of principal component coefficient scores in the first principal component, we determined the coefficients for the z-scores of CSF TDP-43, CSF NfL, and plasma NfL as 0.689, 0.841, and 0.670 (namely 1: 1.22: 0.97), respectively. We generated the optimal composite biomarkers using the weight: (1.22 x z-score of CSF NfL) + (1 x z-score of CSF TDP-43), (1.22 x z-score of CSF NfL) + (0.97 x z-score of plasma NfL), and (0.97 x z-score of plasma NfL) + (1x z-score of CSF TDP-43). AUC values are indicated in the graphs. The title of each graph represents the composite biomarkers used as an independent variable on analysis: (A): the composite biomarker of (1.22 x z-score of CSF NfL) + (1 x z-score of CSF TDP-43) in the discovery cohort. The red and blue dotted lines respectively indicate the ROC curves of CSF NfL alone and CSF TDP 43 alone for reference (see Supplementary Figs. 1 and 3 in Supplementary Files 2 and 4 regarding the ROC analyses of each biomarker for details.) (B): the composite biomarker of (1.22 x z-score of CSF NfL) + (0.97 x z-score of plasma NfL) in the discovery cohort. The red and blue dotted lines respectively indicate the ROC curves of CSF NfL alone and plasma NfL alone. (C): the composite biomarker of (0.97 x z-score of plasma NfL) + (1x z-score of CSF TDP-43. The red and blue dotted lines respectively indicate the ROC curves of CSF TDP-43 alone and plasma NfL alone. (D): the composite biomarker of (1.22 x z-score of CSF NfL) + (1 x z-score of CSF TDP-43) in the validation cohort. The red and blue dotted lines respectively indicate the ROC curves of CSF NfL alone and TDP-43 alone.

1. Van den Bergh PY, Hadden RD, Bouche P, et al. European Federation of Neurological Societies/Peripheral Nerve Society guideline on management of chronic inflammatory demyelinating polyradiculoneuropathy: report of a joint task force of the European Federation of Neurological Societies and the Peripheral Nerve Society - first revision. Eur J Neurol. 2010 Mar;17(3):356-63.

2. Fokke C, van den Berg B, Drenthen J, Walgaard C, van Doorn PA, Jacobs BC. Diagnosis of Guillain-Barre syndrome and validation of Brighton criteria. Brain. 2014 Jan;137(Pt 1):33-43.

3. PNS JTFotEat. European Federation of Neurological Societies/Peripheral Nerve Society guideline on management of multifocal motor neuropathy. Report of a joint task force of the European Federation of Neurological Societies and the Peripheral Nerve Society--first revision. J Peripher Nerv Syst. 2010 Dec;15(4):295-301.

4. Needham M, Mastaglia FL. Inclusion body myositis: current pathogenetic concepts and diagnostic and therapeutic approaches. Lancet Neurol. 2007 Jul;6(7):620-31.
